# Chemokine receptor 5 blockade modulates macrophage trafficking in renal ischaemic‐reperfusion injury

**DOI:** 10.1111/jcmm.15207

**Published:** 2020-03-30

**Authors:** Kyung Don Yoo, Ran‐hui Cha, Sunhwa Lee, Ji Eun Kim, Kyu Hong Kim, Jong Soo Lee, Dong Ki Kim, Yon Su Kim, Seung Hee Yang

**Affiliations:** ^1^ Department of Internal Medicine Ulsan University Hospital University of Ulsan College of Medicine Ulsan Korea; ^2^ Department of Internal Medicine National Medical Center Seoul Korea; ^3^ Department of Internal Medicine Kangwon National University Hospital Chuncheon Korea; ^4^ Department of Biomedical Sciences College of Medicine Seoul National University Seoul Korea; ^5^ Department of Internal Medicine Korea University Guro Hospital Seoul Korea; ^6^ Department of Internal Medicine Seoul National University Hospital Seoul Korea; ^7^ Department of Internal Medicine Seoul National University College of Medicine Seoul Korea; ^8^ Kidney Research Institute Seoul National University Seoul Korea; ^9^ Biomedical Research Institute Seoul National University Hospital Seoul Korea

**Keywords:** acute kidney injury, bilateral ischaemia‐reperfusion injury, CC chemokine receptor 5, chemokine, macrophage

## Abstract

Chemokine receptor 5 (CCR5) is a pivotal regulator of macrophage trafficking in the kidneys in response to an inflammatory cascade. We investigated the role of CCR5 in experimental ischaemic‐reperfusion injury (IRI) pathogenesis. To establish IRI, we clamped the bilateral renal artery pedicle for 30 min and then reperfused the kidney. We performed adoptive transfer of lipopolysaccharide (LPS)‐treated RAW 264.7 macrophages following macrophage depletion in mice. B6.CCR5^−/−^ mice showed less severe IRI based on tubular epithelial cell apoptosis than did wild‐type mice. CXCR3 expression in CD11b^+^ cells and inducible nitric oxide synthase levels were more attenuated in B6.CCR5^−/−^ mice. B6.CCR5^−/−^ mice showed increased arginase‐1 and CD206 expression. Macrophage‐depleted wild‐type mice showed more injury than B6.CCR5^−/−^ mice after M1 macrophage transfer. Adoptive transfer of LPS‐treated RAW 264.7 macrophages reversed the protection against IRI in wild‐type, but not B6.CCR5^−/−^ mice. Upon knocking out CCR5 in macrophages, migration of bone marrow‐derived macrophages from wild‐type mice towards primary tubular epithelial cells with recombinant CCR5 increased. Phospho‐CCR5 expression in renal tissues of patients with acute tubular necrosis was increased, showing a positive correlation with tubular inflammation. In conclusion, CCR5 deficiency favours M2 macrophage activation, and blocking CCR5 might aid in treating acute kidney injury.

## 1 INTRODUCTION

Renal ischaemic‐reperfusion injury (IRI) is a complicated orchestrated event that elicits diverse immunological responses. Monocytes/macrophages, which exhibit great pliability, are important components of renal IRI. Their presence in the kidneys is closely correlated with a loss of renal function,^1^, ^2^, ^3^ and plasticity of macrophages affects the incidence of acute renal damage owing to chronic fibrosis.^4^, ^5^, ^6^

Chemokine receptor 5 (CCR5) is a G protein‐coupled receptor that spans seven transmembrane domains and a co‐receptor for macrophage‐trophic human immunodeficiency virus (HIV) type 1 strains.^7^ CCR5 is mainly associated with organ development, including angiogenesis, haematopoiesis, metastasis and chemotaxis. It is encoded on chromosome 3p21 and expressed by various immune cells such as resting T lymphocytes that have memory and effector T‐cell phenotypes, monocytes, macrophages and immature dendritic cells.^7^ Several ligands, including RANTES (regulated on activation, normal T cell expressed and secreted/CCL5), monocyte chemo‐attractant protein 1 (MCP‐1), macrophage inflammatory protein (MIP)‐1α and MIP‐1β, react with CCR5, are activated by CCR5 retroactive to CCR5 ligands.

CCR5 signalling plays various roles in inflammation and chemokine receptor expression because of macrophage heterogeneity.^8^ A distinction between M1 and M2 macrophages suggests that the initiation and response to lipopolysaccharide (LPS)‐ or IFN‐γ‐induced stimulation are dependent on different signalling pathways of the Th1 or Th2 response. M2 macrophages are quite different from classically activated macrophages that produce trophic amines.^9^, ^10^ Phenotypic diversity of macrophages is important in acute ischaemic kidney injury development^2^, ^3^ and progression to chronic kidney disease.^4^ Interestingly, several cytokines and chemokines are involved in the differentiation, recruitment and migration of monocytes and macrophages during this process.^11^, ^12^ CCR2 and MCP‐1 play key roles in macrophage heterogenicity and plasticity,^13^ but insufficient data are available on the association between CCR5 and the origin and subsets of macrophages. Moreover, data from post‐transplantation biopsies show conflicting results because M2 macrophage deposition occurs during pro‐inflammatory reactions rather than during tissue repair,^14^, ^15^ indicating that further research is necessary.

Here, we aimed to determine (a) the effect of macrophage phenotype on the expression of CCR5 and other chemokines, (b) the influence of macrophage phenotype on CCR5 signalling inhibition, and (c) the relevance of the CCR5 signalling pathway to IRI using in vivo and in vitro models. Finally, we analysed post‐transplantation kidney biopsies to clarify the association between CCR5 and macrophages in acute kidney injury and clinical outcomes.

## 2 MATERIALS AND METHODS

All experiments were performed with the approval of the Institutional Animal Care and Use Committee of the Clinical Research Institute of Seoul National University Hospital and in accordance with the Guidelines for the Care and Use of Laboratory Animals of the National Research Council. All experiments dealing with human specimens were also approved by the institutional review board of our institution (IRB number: H1910‐011‐1067). The experimental methods used in our study have been previously described.^16^, ^17^, ^18^, ^19^ All experiments were conducted in accordance with the guidelines of the 2013 Declaration of Helsinki.

### 2.1 Experimental animals

Male, 8‐week‐old, C57BL/6 (B6) mice were purchased from Orient Company. B6.CCR5^−/−^ mice were originally produced by the Jackson Laboratory. All mice were raised in a pathogen‐free animal facility. For extended methods related to in vitro and in vivo manipulations, including induction of renal ischaemic‐reperfusion injury (IRI), histologic analysis, confocal microscopy, quantitative real‐time PCR, Western blot, cytokine assay, flow cytometry, mouse tubular epithelial cell and bone marrow‐derived cell isolation, culture, in vitro cell migration assay, and statistical methods, as well as the functional study protocol and ischaemia‐induced hypoxic condition procedures, please refer to online supplemental data.

## 3 RESULTS

### 3.1 Depletion of CCR5 attenuates ischaemia‐reperfusion injury

**FIGURE 1:**
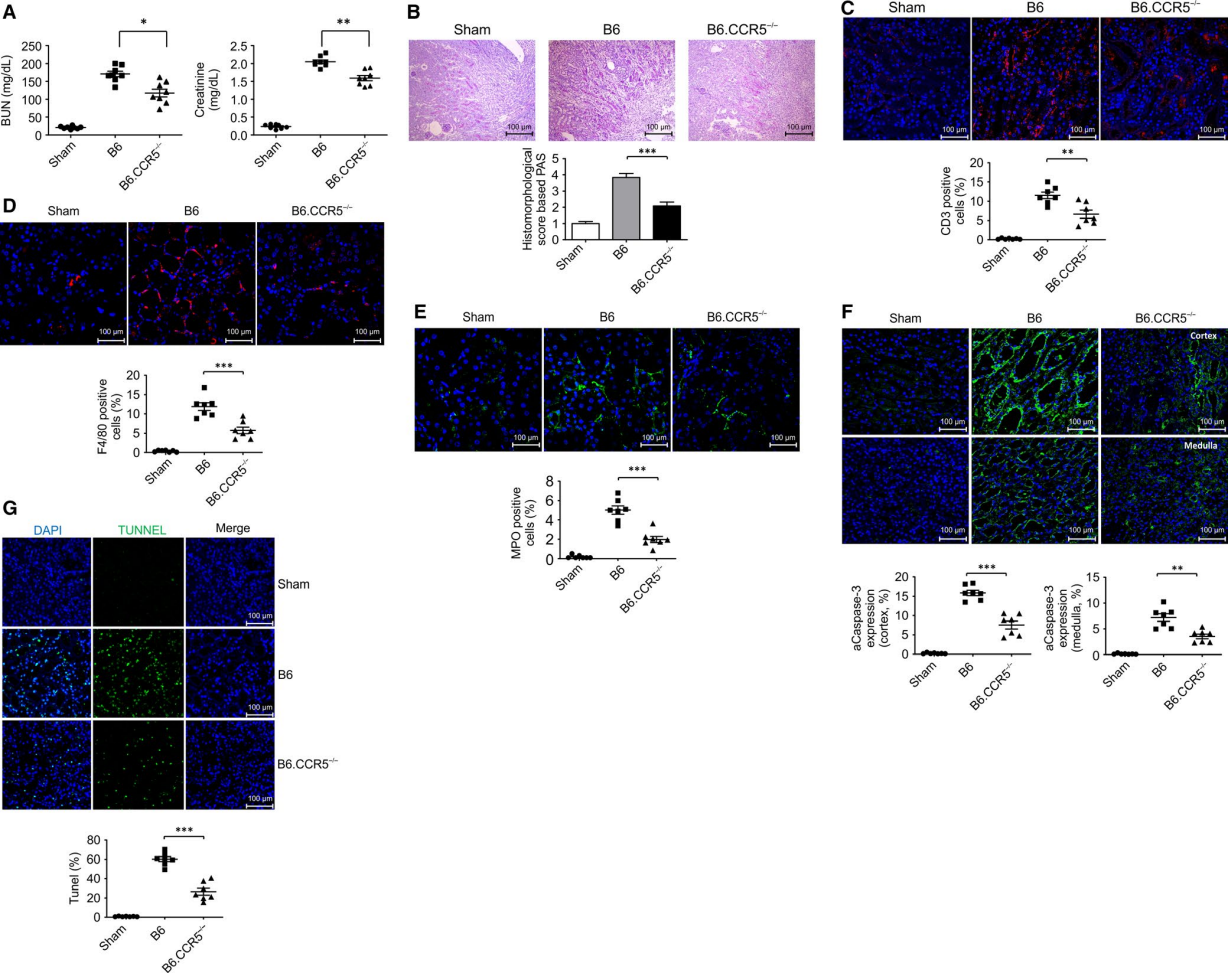
The role of CCR5 regulation in kidney IRI. A, Comparisons between baseline kidney function in mice with CCR5 knockout and control mice after inducing IRI in the laboratory; blood urea nitrogen, creatinine, (B) PAS statin, (C) CD3, (D) F4/80, (E) MPO and (F) caspase 3 were detected using confocal microscopy. CCR5 null mice showed less extensive pathological changes and expressions of CD3, F4/80, MPO and caspase 3 than did B6 WT mice (n = 8/group; *P < .05, **P < .01). These results represent one of three independent experiments

To determine the specific role of CCR5 in IRI in the kidney, we induced renal ischaemia in mice. Renal function deteriorated significantly after IRI in the sham control and wild‐type disease control mice (Cr 0.46 ± 0.03 mg/dL vs 2.05 ± 0.05 mg/dL; ***P < .001). In B6.CCR5‐deficient (B6.CCR5^−/−^) mice, however, the severity of renal dysfunction was less than that in wild‐type B6 mice (Cr 2.05 ± 0.05 mg/dL vs 1.59 ± 0.07 mg/dL; **P < .01) (Figure 1A). This pattern was also observed in the level of blood urea nitrogen (BUN) (BUN 170.8 ± 7.70 mg/dL vs 117.6 ± 10.85 mg/dL; *P < .05).

IRI‐induced tubular necrosis involves the disruption of tubular epithelial cells. The damage was more extensive in wild‐type than in B6.CCR5^−/−^ mice (Figure 1B), and the number of CD3‐ (Figure 1C), F4/80‐ (Figure 1D), MPO‐ (Figure 1E), caspase‐3‐ (Figure 1F), and TUNEL‐ (Figure 1G) positive cells was much lower in B6.CCR5^−/−^ than in wild‐type mice. These histological changes were consistent with the functional data.

### 3.2 Effects of CCR5 deficiency and changes in cytokine milieu

**FIGURE 2:**
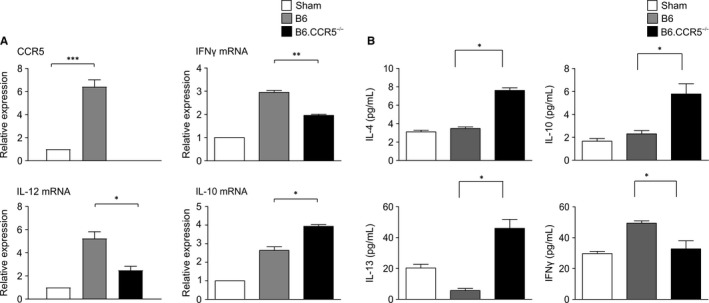
The effect of CCR5 deficiency in the IRI model. A, The mRNA levels of inflammatory cytokines. The results of real‐time PCR revealed that the expressions of various inflammatory cytokines, including CCR5 and IFN‐γ, were significantly elevated in wild‐type mice compared with those in control mice and were significantly attenuated in B6.CCR5^−/−^ mice (**P < .01). B, Whole kidney protein extract assay. On ELISA, the expressions of Th2 dominant cytokines, including IL‐4, IL‐10 and IL‐13, were significantly elevated in B6.CCR5^−/−^ mice compared with those in control mice and were significantly attenuated in wild‐type mice (n = 8 per each group, *P < .05 and **P < .01)

To examine how patterns of cytokine expression affect the extent of renal damage caused by IRI, we quantified mRNA levels using real‐time PCR 24 hours after induction of IRI. Pro‐inflammatory cytokines and chemokines such as interferon‐γ (IFN‐γ), IL‐12 and CCR5 were increased by IRI in wild‐type mice. However, in B6.CCR5^−/−^ mice, pro‐inflammatory cytokines were significantly suppressed. Although IRI induced an increase in pro‐inflammatory cytokines in wild‐type mice, B6.CCR5^−/−^ mice showed enhanced expression of regulatory cytokines (IL‐10) during IRI (Figure 2A). Enzyme‐linked immunosorbent assay (ELISA), performed on whole kidney protein extracts, showed that IL‐4, IL‐10 and IL‐13, but not IFN‐γ, were increased in response to IRI in CCR5^−/−^ mice (Figure 2B).

### 3.3 Decreased T‐cell infiltration in B6.CCR5^−/−^ compared to that in wild‐type mice

**FIGURE 3:**
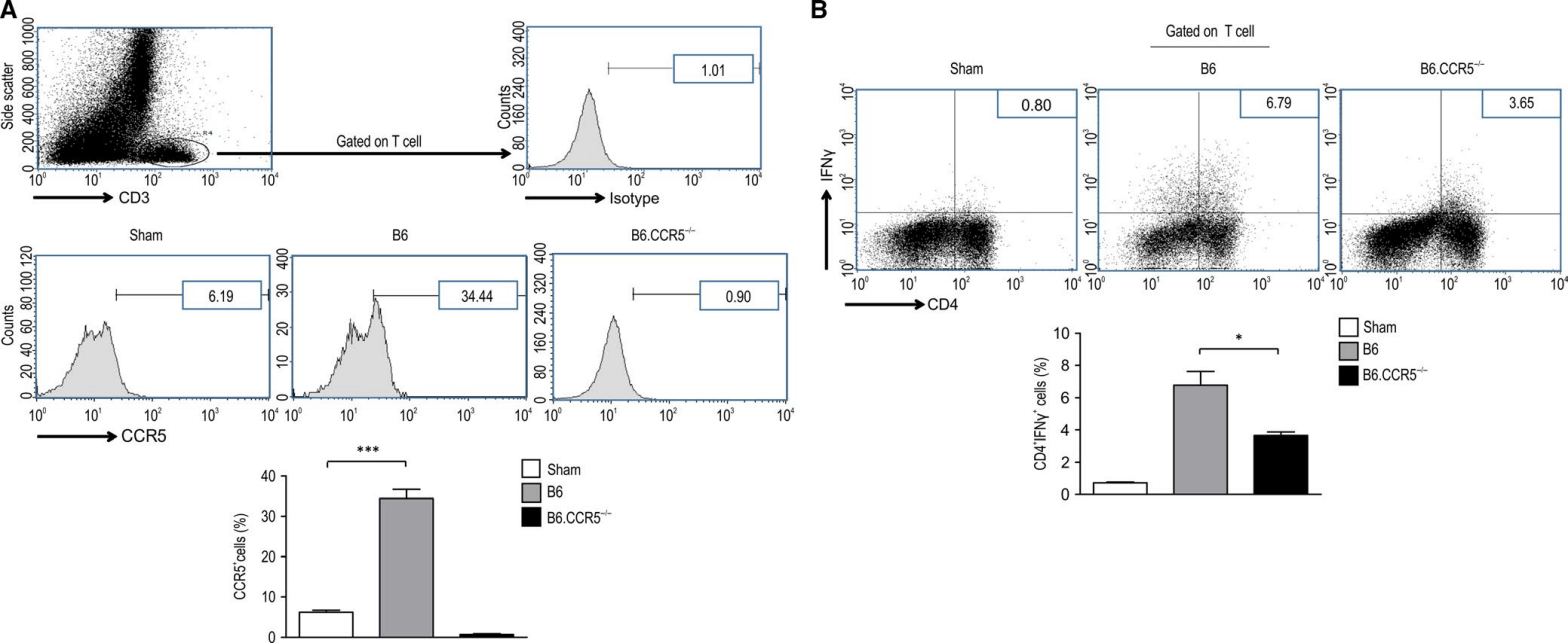
CCR5 inhibition significantly decreases damage in the kidney due to ischaemia‐reperfusion injuries from T lymphocytes. A, Representative flow cytometry images. Flow cytometry analysis of CCR5 expression on CD3‐positive T cells isolated from the kidneys of wild‐type mice and CCR5‐deficient mice 6 h after IRI. Values are represented as the means ± SEM of the cell counts from the kidney samples from 3 to 4 mice in each group. (*** *P* < .001). The proportions of intrarenal CCR5 expression gated on T cells in each animal group were as follows: 6.19 ± 0.47% in control mice, 34.44 ± 2.25% in wild‐type mice and 0.90% in B6.CCR5^−/−^ mice. B, The proportions of intrarenal IFN‐γ expression gated on T cells in each animal group were as follows: 6.79 ± 0.82% in wild‐type mice and 3.65 ± 0.21% in B6.CCR5^−/−^ mice

Fluorescence‐activated cell sorting (FACS) staining of intrarenal lymphocytes for CCR5 expression showed that trafficking increased significantly in wild‐type mice relative to that in the sham mice and isotype controls (34.44 ± 2.25% vs 6.19 ± 0.47%, ***P < .001) (Figure 3A). In sham mice, IFN‐γ was expressed in 0.80% of the intrarenal CD4 T cells; furthermore, IRI induced an increase in intrarenal IFN‐γ in wild‐type mice, but attenuated this cytokine in B6.CCR5^−/−^ mice (6.79 ± 0.82% vs 3.65 ± 0.21) (Figure 3B) (n = 3 for each animal group).

### 3.4 Macrophage and CCR5 crosstalk

**FIGURE 4:**
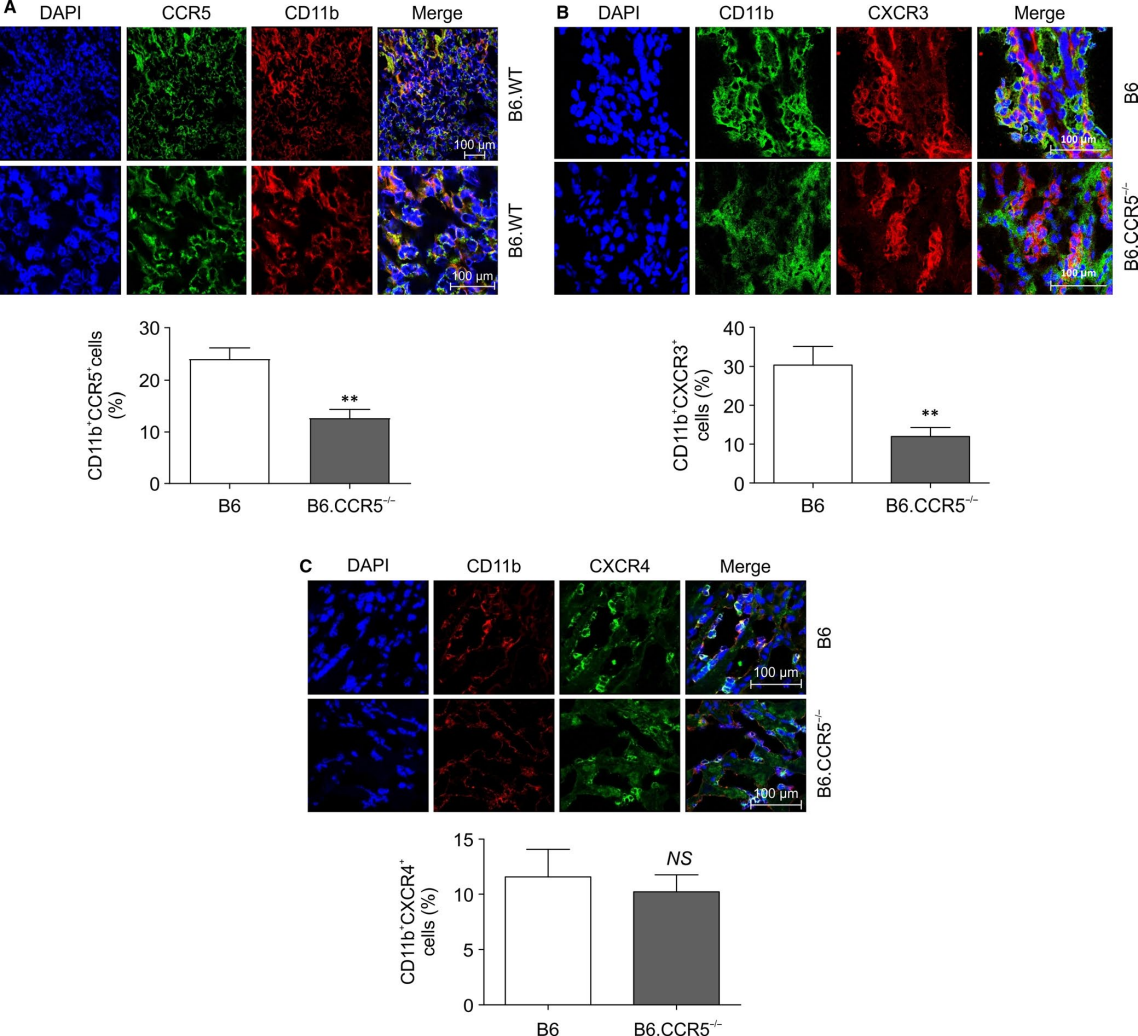
The crosstalk of macrophages and CCR5. A, The expression of CD11b^+^/CCR5^+^ in wild‐type mice after inducing IRI for immunofluorescence staining on confocal microscopy (magnification: ×400 [top], ×1000 [bottom]). B, The expression of CD11b^+^/CXCR3^+^ in wild‐type mice after inducing IRI visualized by immunofluorescence staining using a confocal microscope. Tissue CD11b^+^/CXCR3^+^ expression was enhanced in wild‐type mice and ameliorated in B6.CCR5^−/−^ mice, as determined by immunofluorescence staining (magnification: ×1000 [top/bottom]). Cell counts for labelled CD11b (green) and labelled CXCR3 cells (red) were determined by fluorescence microscopy. Values are represented as the means ± SEM of cell counts per 10 high‐power fields (hpf) per kidney from five mice in each group (CD11b^+^/CXCR3^+^ cells, 30.35 ± 4.768 in wild‐type mice vs 11.99 ± 2.324 in B6.CCR5^−/−^; **P < .01). C, The expression of CD11b^+^/CXCR4^+^ in wild‐type mice after inducing IRI visualized by immunofluorescence staining on a confocal microscope. Tissue CD11b^+^/CXCR4^+^ expression was enhanced in wild‐type mice and slightly decreased in B6.CCR5^−/−^ mice as determined by immunofluorescence staining (magnification: ×1000 [top/bottom]). (CD11b^+^/CXCR4^+^ cells, 11.58 ± 2.48 in wild‐type mice vs 10.22 ± 1.54 in B6.CCR5^−/−^; *P* = .655)

B6.CCR5^−/−^ mice showed less aggravated IRI in terms of apoptosis of tubular epithelial cells and creatinine (Cr) concentrations than did B6 wild‐type mice. T‐cell and macrophage infiltration decreased in B6.CCR5^−/−^ compared with that in wild‐type mice. Next, we assessed whether monocytes and macrophages migrated to the inflammation site and were induced by IRI. After IRI, CD11b^+^CCR5^+^ cells were commonly expressed in wild‐type mice and could be tracked by confocal microscopy (Figure 4A). CXCR3 and CXCR4 are co‐receptors of CCR5. Upon injury, CD11b^+^CXCR3^+^ cells were attenuated in B6.CCR5^−/−^ compared with that in wild‐type mice (Figure 4B). This pattern was similar to that in CD11b^+^CXCR4^+^ cells (Figure 4C). In this IRI model, CXCR3 infiltration was more prominent than CXCR4 infiltration upon confocal microscopy examination.

### 3.5 CCR5 modulation of macrophage phenotype

**FIGURE 5:**
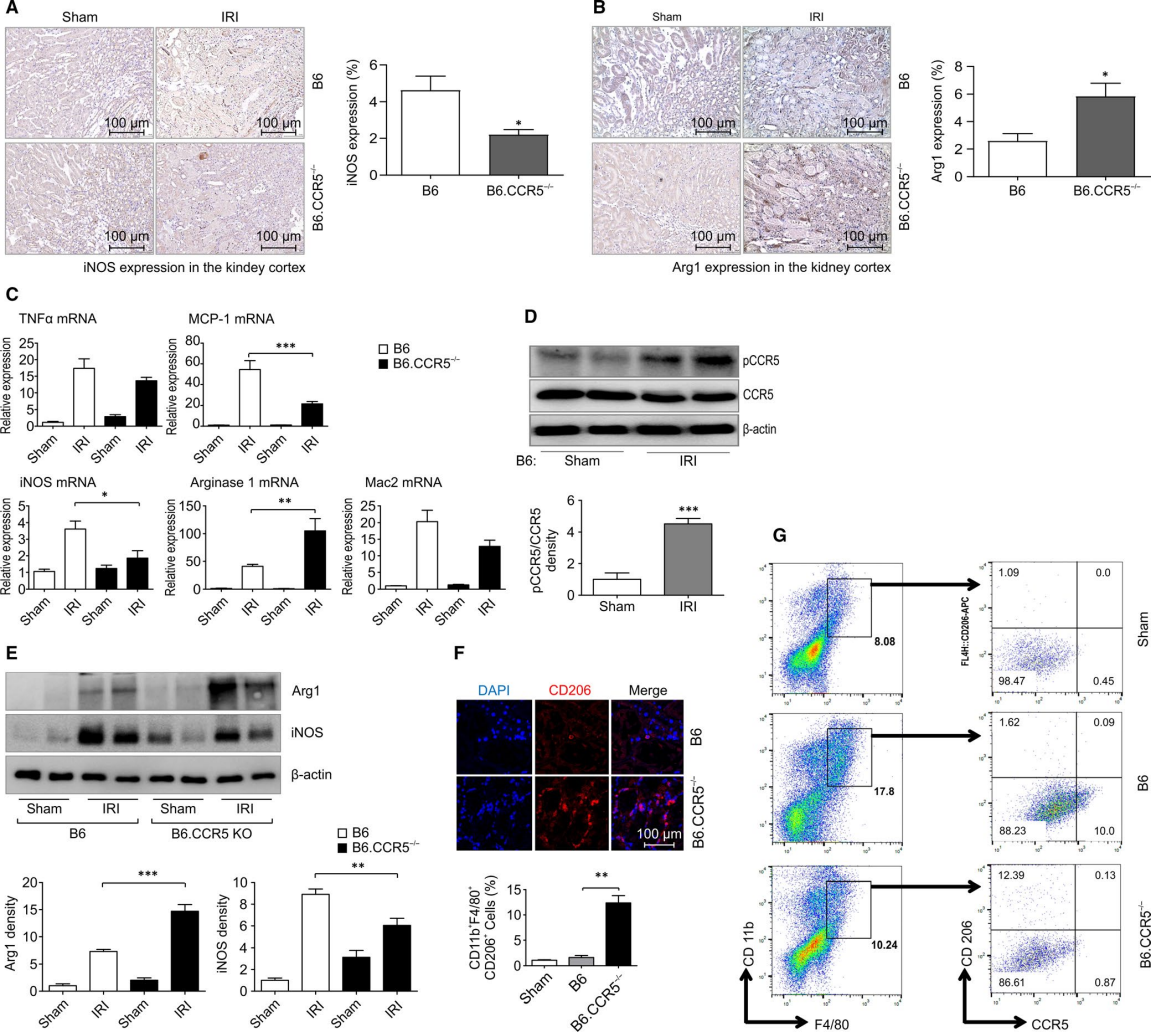
The expression of macrophage heterogeneity in injured B6.CCR5^−/−^ mice. A, The classical activation of M1 macrophages was representative of enzyme activity after inducing IRI; immunohistochemistry stain for iNOS was increased in wild‐type mice. B, The immunohistochemistry stain for arginase‐1 was intensified in B6.CCR5^−/−^ mice compared to that in B6 WT mice. C, The results of real‐time PCR revealed that the mRNA levels of Th1 inflammatory cytokines such as MCP‐1 and iNOS were significantly decreased in B6.CCR5^−/−^ mice compared to those in wild‐type mice (***P < .001, n = 8 for each group). D, The protein levels of CCR5 were increased in wild‐type mice after inducing IRI. E, In Western blot densitometry analyses, the expressions of M2 macrophage markers, such as CD206 and arginase‐1, were substantially elevated in B6.CCR5^−/−^ mice and attenuated in wild‐type mice. F, The comparison of expression for CD206 immunofluorescence staining on confocal microscopic examination. G, Representative flow cytometry images for CD206^+^ CCR5^+^ in CD11b^+^ F4/80^+^ cells. The induction of CD206^+^ in CD11b^+^F4/80^+^ cells was increased in KO mice compared with that in wild‐type mice. (**P < .01, n = 3 for each group)

Classical activation of M1 macrophages caused enzymes such as inducible nitric oxide synthase (iNOS), which produces nitric oxide from arginine, to be attenuated in B6.CCR5^−/−^ compared with that in wild‐type mice (Figure 5A). Arginase‐1 is a marker of M2 macrophages that metabolizes l‐arginine through a non‐fungicidal pathway and is involved in tissue repair during an inflammation cascade. Using immunofluorescence, arginase‐1 stained more intensely in B6.CCR5^−/−^ than in wild‐type mice (Figure 5B). The intrarenal mRNA expression of pro‐inflammatory cytokines such as TNF‐α, IFN‐γ and MCP‐1 decreased, whereas arginase‐1 expression increased after IRI in B6.CCR5^−/−^ mice (Figure 5C). CCR5 protein levels increased in wild‐type mice after induction of IRI (Figure 5D). The M2 macrophage phenotype markers, arginase‐1 and CD206, both increased in B6.CCR5^−/−^ compared with that in wild‐type mice (Figure 5E). Moreover, with CCR5 deficiency, there was a noteworthy increase in CD206^+^ macrophages (Figure 5F). CD206^+^CD11b^+^F4/80^+^ cells were induced in both wild‐type and CCR5 knockout mice after IRI. The proportions of intrarenal CD206^+^ macrophages in each group were as follows: 1.09 ± 0.09% in control mice, 1.62 ± 0.40% in wild‐type mice and 12.39 ± 1.43% in B6.CCR5^−/−^ mice (Figure 5G).

### 3.6 CCR5 inhibition and amelioration of hypoxia‐induced injury

**FIGURE 6:**
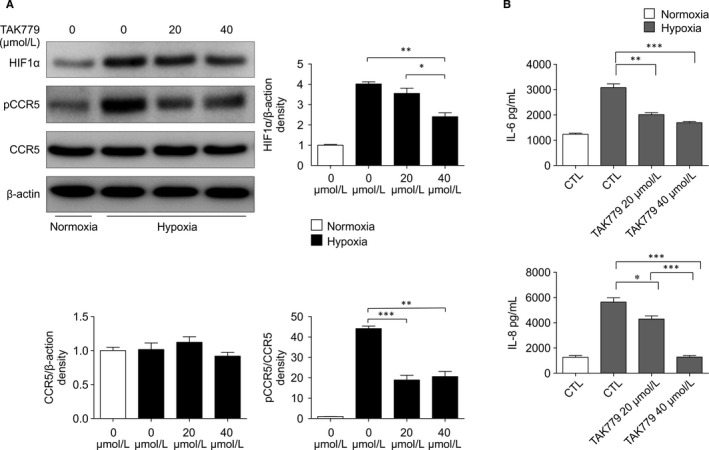
The expression of p‐CCR5 under in vitro hypoxia in HK2 cells. A, For in vitro studies, hypoxic injury for 6 h resulted in phospho‐CCR5 expression. Moreover, the expression of phospho‐CCR5 under hypoxia was markedly attenuated by the administration of TAK779, which attenuated HIF‐1α expression in HK‐2 cells in a dose‐dependent manner. B, Protein levels of IL‐6 and IL‐8 were increased in hypoxic condition, and administration of TAK779 attenuated the increase in a dose‐dependent manner. Values are represented as the means ± SEM of ELISA assay per kidney in three mice in each group (IL‐6, 3082 ± 147 pg/mL in 6 h hypoxia vs 2013 ± 76.29 pg/mL in 6 h hypoxia + TAK779 20 μmol/L vs 1694 ± 46.07 pg/mL + TAK779 40 μmol/L; IL‐8, 5643 ± 342.3 pg/mL in 6 h hypoxia vs 4295 ± 246.0 pg/mL in 6 h hypoxia + TAK779 20 μmol/L vs 1282 ± 117.7 pg/mL + TAK779 40 μmol/L)

To demonstrate further the role of CCR5 inhibition in hypoxia, we conducted in vitro experiments using tubular epithelial cell lines. We confirmed the expression of CCR5, and not HIF‐1α or phospho (p)‐CCR5, in normoxic conditions in human kidney‐2 (HK‐2) cells (Figure 6A). Simultaneously, we cultured HK‐2 cells in hypoxic conditions with TAK779. Six hours after hypoxic injury, p‐CCR5 was expressed. A CCR5‐CXCR3 inhibitor attenuated these changes in expression in a dose‐dependent manner (Figure 6A). Supernatant protein was quantified using ELISA to measure IL‐6 and IL‐8 levels (Figure 6B). Hypoxia increased IL‐6 and IL‐8 protein levels, and TAK779 attenuated this increase in a dose‐dependent manner.

### 3.7 RAW 264.7 macrophage stimulation and adoptive transfer into mice

**FIGURE 7:**
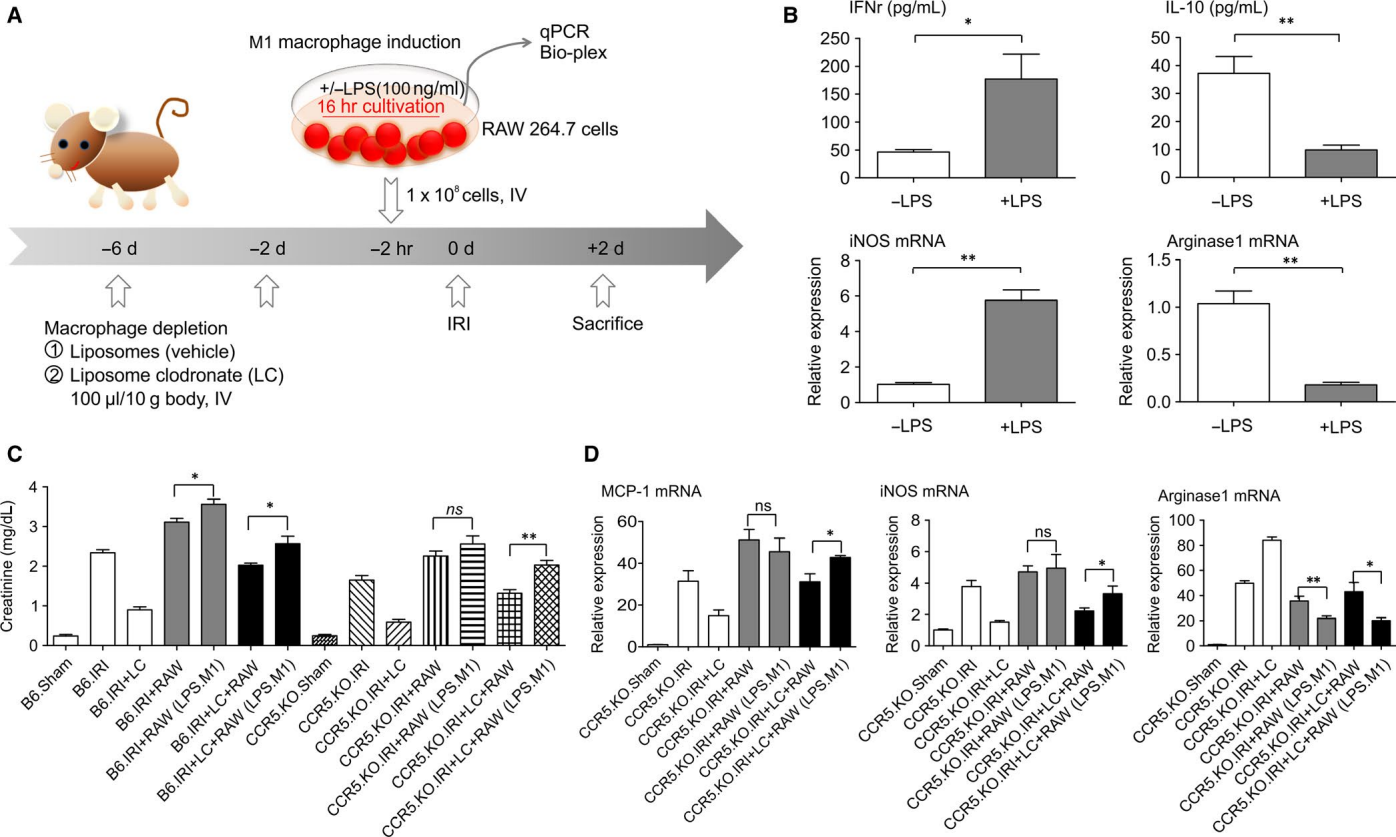
In vitro‐cultured RAW 264.7 macrophage cells and adoptive transfer to wild‐type and B6.CCR5^−/−^ mice in the IRI model. A, Detailed schedule for in vitro‐cultured RAW 264.7 macrophage cells and adoptive transfer has been presented. B, The M1 macrophage tendency was induced by stimulating RAW 264.7 cells with LPS, and 1 × 10^8^ cells were adoptively transferred 2 h before the induction of IRI. The amplitude of the expression of INF‐γ and iNOS was increased, which indicates that they reflect the characteristics of M1 macrophage subpopulation and the adoptive transfer of these macrophages. C, In both wild‐type and B6.CCR5^−/−^ mice, injury was decreased after LC treatment (white bars). Undergoing the adoptive transfer of RAW cells with or without LPS showed an increase in the injury in wild‐type mice, whereas there was no difference in the degree of injuries in B6.CCR5^−/−^ mice (grey bars). Ultimately, LC‐pre‐treated RAW cells with or without LPS were adoptively transferred, and injury increased in both wild‐type and B6.CCR5^−/−^ mice, but predominantly in the latter (black bars). The detrimental effect of RAW cell adoptive transfer was weakened in B6.CCR5^−/−^ mice. D, The adoptive transfer of M1 macrophages decreased the mRNA expression of pro‐inflammatory cytokines, such as MCP‐1 and iNOS, in B6.CCR5^−/−^ mice (n = 5 per each group, *P < .05 and **P < .01)

To investigate further the role of macrophage‐ and CCR5‐signalling in IRI, LPS‐treated RAW 264.7 macrophages were adoptively transferred into mice. RAW 264.7 macrophages were cultured with or without LPS (100 ng/mL) for 16 hours. B6 wild‐type and B6.CCR5^−/−^ mice were treated with liposomal clodronate (LC) (100 μL/10 g, intravenous) to deplete macrophages for 6 days to induce IRI (Figure 7A). ELISA of supernatants showed that IFN‐γ protein (RAW 264.7 macrophages with LPS 177.2 ± 44.38 pg/mL, without LPS 46.55 ± 4.40 pg/mL; *P < .05) and iNOS mRNA (RAW 264.7 macrophages with LPS 5.76 ± 0.58 pg/mL, without LPS 1.03 ± 0.10 pg/mL; **P < .01) expression increased in LPS‐treated RAW 264.7 macrophages, which tended to induce M1 macrophages (Figure 7B).

As expected, in both wild‐type and B6.CCR5^−/−^ mice, LC macrophage depletion showed a protective effect without the adoptive transfer of RAW 264.7 macrophages post‐IRI induction (B6, Cr 2.34 ± 0.17 mg/dL vs B6 + LC, Cr 0.89 ± 0.17 mg/dL; B6.CCR5^−/−^, Cr 1.65 ± 0.24 mg/dL vs B6.CCR5^−/−^ + LC, Cr 0.59 ± 0.14 mg/dL). However, in native macrophage conditions, adoptive transfer to RAW 264.7 macrophage‐ and LPS‐treated mice aggravated renal damage after IRI induction in wild‐type mice but not in B6.CCR5^−/−^ mice (B6 + RAW 264.7 macrophages, Cr 3.02 ± 0.13 mg vs B6 + RAW 264.7 macrophages + LPS, Cr 3.56 ± 0.28 mg/dL; B6.CCR5^−/−^ + RAW 264.7 macrophages, Cr 2.25 ± 0.28 mg/dL vs B6.CCR5^−/−^ + RAW 264.7 macrophages + LPS, Cr 2.56 ± 0.43 mg/dL) (Figure 7C). In vivo administration of RAW 264.7 macrophages and LC for macrophage depletion with LPS after IRI induction aggravated renal damage more than RAW 264.7 macrophages and LC without LPS in both groups. Interestingly, renal impairment resulting from adoptive transfer of RAW 264.7 macrophages was more severe in B6.CCR5^−/−^ mice than in wild‐type mice (B6 + RAW 264.7 macrophages + LC, Cr 2.02 ± 0.11 mg/dL, B6 + RAW 264.7 macrophages + LC +LPS, Cr 2.57 ± 0.40 mg/dL; B6.CCR5^−/−^ + RAW 264.7 macrophages + LC, Cr 1.32 ± 0.19 mg/dL vs B6.CCR5^−/−^ + RAW 264.7 macrophages + LC +LPS, Cr 2.03 ± 0.26 mg/dL) (Figure 7C).

Finally, B6.CCR5^−/−^ mice showed less severe injuries than wild‐type mice after the transfer of RAW 264.7 macrophages, regardless of LPS pre‐treatment without macrophage depletion (B6 + RAW 264.7 macrophages, Cr 3.02 ± 0.13 mg/dL vs B6.CCR5^−/−^ + RAW 264.7 macrophages, Cr 2.25 ± 0.28 mg/dL; B6 + RAW 264.7 macrophages + LPS, Cr 3.56 ± 0.28 mg/dL vs B6.CCR5^−/−^ + RAW 264.7 macrophages + LPS, Cr 2.56 ± 0.43 mg/dL) (Figure 7C). Adoptive transfer of LPS‐treated RAW 264.7 macrophages with constitutive expression of iNOS reversed functional protection against IRI in wild‐type mice, but not in B6.CCR5^−/−^ mice. Further, adoptive transfer of M1 macrophages decreased mRNA expression of pro‐inflammatory cytokines such as MCP‐1 and iNOS in B6.CCR5^−/−^ mice (Figure 7C,D).

### 3.8 CCR5 modulation of activated macrophage migration

**FIGURE 8:**
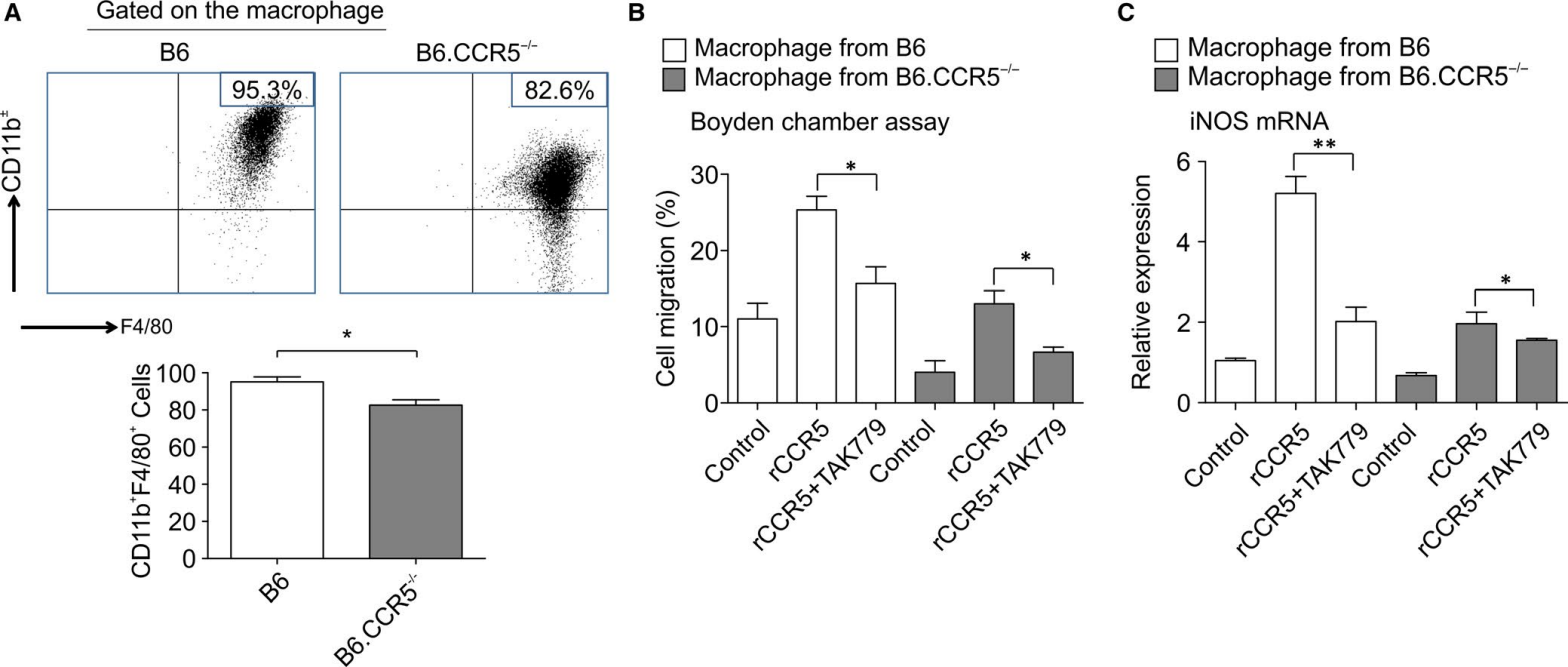
The macrophage chemotaxis test. A, The FACS staining of gating on intrarenal macrophages showed a significant increase in CD11b^+^F4/80^+^ in wild‐type mice compared with that in B6.CCR5^−/−^ mice. B, The results of a cell migration test on wild‐type and B6.CCR5^−/−^ mice. Recombinant CCR5 treatment resulted in a >20% increase in the migration and the CCR5 antagonist TAK779 treatment resulted in a significant decrease. A similar pattern was observed in B6.CCR5^−/−^ mice. C, A comparison of the cell mRNA level of iNOS between wild‐type and B6.CCR5^−/−^ mice. The degree of mRNA expression of iNOS followed the same pattern as the degree of migration

FACS staining gated on intrarenal macrophages showed a significant increase in CD11b^+^F4/80^+^ cells in wild‐type compared with that in B6.CCR5^−/−^ mice (95.3 ± 2.47% vs 82.6 ± 2.94%). (Figure 8A). Moreover, in vitro macrophage migration assays were performed to clarify the effect of M1 macrophages on the chemotaxis of CCR5‐deficient cells using a Boyden chamber with a filter membrane with 8‐μm pores. The migration of BMDMs towards primary tubular epithelial cells with recombinant CCR5 was greater in wild‐type mice by 25% than in B6.CCR5^−/−^ mice. Additionally, blockade of CCR5 by TAK779 inhibited macrophage migration (Figure 8B). In a co‐culture system of tubular epithelial cells and macrophages, macrophages induced iNOS mRNA expression (Figure 8C).

### 3.9 Increased CCR5 expression in the kidney biopsy samples

**FIGURE 9:**
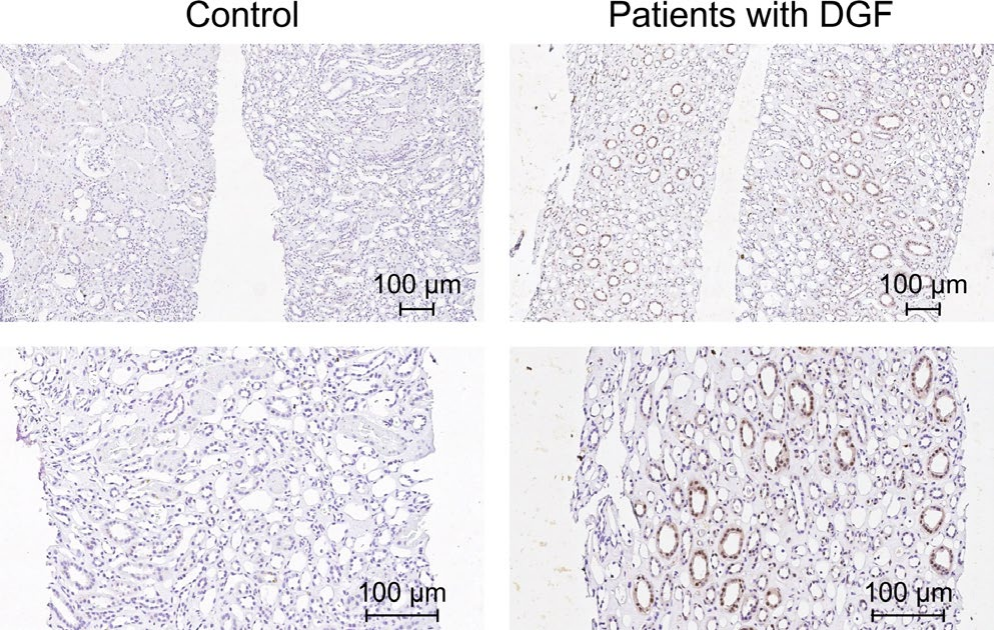
Increased CCR5 expression in the kidney biopsy samples. A, Increased CCR5 expression in a kidney transplant recipient with delayed graft function compared with that in the normal kidney sample on immunohistochemistry staining (magnification: ×40 [top], ×100 [bottom])

The kidney biopsy tissues from kidney transplant recipients with delayed graft function were assessed for biopsy‐proven acute tubular necrosis (ATN) without evidence of cellular or humoral rejection. Those with ATN were used to evaluate whether phosphorylation of CCR5 had a detrimental effect on IRI in patients undergoing transplantation. ATN in renal tissues from patients with glomerular nephritis was also examined (Table 1). The characteristics of the 25 patients included in this analysis are shown in Table 1. We analysed p‐CCR5 in renal biopsy samples from 14 patients who underwent transplantation (Table 1: case numbers 1‐14), 11 patients with glomerulonephritis (Table 1: case numbers 15‐25), and normal controls (Table 1: case numbers 26‐31). Numbers of p‐CCR5 cells increased relative to those in the normal control sample (Figure 9); in normal controls, p‐CCR5 was scarcely detected. By immunohistochemical staining morphometry, kidney transplant recipients were more likely to have p‐CCR5 cells than patients with glomerulonephritis (5.21 ± 1.32 vs 3.87 ± 1.71, *P* = .038). Renal tissue of patients with renal biopsy (n = 25) frequently contained p‐CCR5 cells, and the number of these cells was positively correlated with tubule and vascular inflammation (tubulitis, *P* = .010, *r* = .508; intimal arteritis, *P* = .017, *r* = .474) (Table 1).

**TABLE 1:** The phosphorylation of CCR5 in the kidney biopsy samples in kidney transplant recipients and patients with glomerulonephritis

Case	Age*/Sex	Primary disease of ESRD	Donor relation	Phosphorylation of CCR5 (%)	GS (%)	I	T	G	V	CT	CI	MM	AH	ESRD development
1	63/M	Unknown	DD	5.80 ± 1.90	5.1	0	0	0	0	0	0	0	0	YES
2	31/M	HTN	DD	4.52 ± 1.23	2.8	0	0	0	0	0	0	0	0	NO
3	53/M	GN	DD	5.74 ± 1.49	7.4	0	0	0	1	0	0	0	1	NO
4	65/M	DM	DD	3.45 ± 0.57	2.4	1	1	0	1	1	1	0	0	NO
5	51/M	DM	DD	3.76 ± 1.44	49.2	1	1	0	0	1	1	0	0	NO
6	55/M	DM	DD	6.51 ± 1.57	6.5	1	1	0	2	1	1	0	2	NO
7	41/F	DM	DD	7.53 ± 0.57	19.0	1	0	0	1	0	0	0	0	NO
8	69/F	DM	DD	6.92 ± 1.06	7.7	1	0	0	2	1	1	0	0	NO
9	57/F	DM	LD	6.31 ± 1.84	33.3	1	0	0	0	0	0	0	0	NO
10	67/M	DM	DD	3.85 ± 1.17	‐	0	0	0	0	0	0	0	0	NO
11	37/F	GN	DD	5.02 ± 2.18	13.6	2	1	0	1	0	0	0	0	NO
12	60/F	PKD	DD	3.65 ± 1.12	6.7	1	0	0	0	0	0	0	0	YES
13	51/F	DM	LD	5.51 ± 2.98	3.6	1	0	0	0	0	0	0	0	YES
14	39/F	Unknown	DD	4.38 ± 1.93	‐	0	0	0	0	0	0	0	0	YES
15	64/M	MCD		4.72 ± 1.50	11.8	1	1	0	0	1	1	0	0	NO
16	62/M	MCD		5.41 ± 1.63	14.3	1	1	0	0	0	0	0	0	NO
17	62/M	MCD		6.67 ± 2.06	11.1	1	1	0	0	0	0	0	0	NO
18	86/M	MN		1.40 ± 1.93	31.4	2	2	0	0	2	1	0	3	YES
19	34/M	MN		2.74 ± 1.10	2.2	1	1	1	0	0	0	0	0	NO
20	78/M	IgAN		3.51 ± 1.86	5.0	1	1	0	0	1	1	0	0	YES
21	23/F	IgAN		5.27 ± 1.11	86.7	2	1	0	0	3	2	0	3	YES
22	55/F	amyloidosis		2.12 ± 0.79	4.9	1	1	0	0	1	1	0	1	NO
23	51/F	MCD		1.84 ± 1.42	40.0	1	1	0	0	0	2	0	0	YES
24	59/F	IgAN		5.21 ± 2.69	6.4	1	1	0	0	1	0	0	1	NO
25	F/53	Unknown		3.78 ± 1.02	11.1	1	1	0	0	1	0	0	0	NO
26	25/F	Control		<0.50	‐	0	0	0	0	0	0	0	0	NO
27	20/M	Control		<0.50	‐	0	0	0	0	0	0	0	0	NO
28	19/M	Control		<0.50	‐	0	0	0	0	0	0	0	0	NO
29	50/F	Control		<0.50	‐	0	0	0	0	0	0	0	0	NO
30	29/M	Control		<0.50	‐	0	0	0	0	0	0	0	0	NO
31	21/M	Control		<0.50	‐	0	0	0	0	0	0	0	0	NO

Note

Pathological finding grade: 0, mild; 1, moderate; 2, severe. Pathological finding description: GS, global sclerosis (%); I, interstitial inflammation; T, tubulitis; G, glomerulitis; V, intimal arteritis; CT, chronic tubular atrophy; CI, chronic interstitial fibrosis; MM, mesangial matrix expansion; AA, arteriolar hyalinosis.

Abbreviations: DD, deceased donor; DM, diabetes mellitus; ESRD, end‐stage renal disease; F, female; GN, glomerular nephritis; HTN, hypertension; Ig AN, immunoglobulin A nephropathy; LD, living donor; M, male; MCD, minimal change disease; MN, membranous nephropathy; PKD, polycystic kidney disease; UPCR, urine protein‐creatinine ratio.

* Age at the time of Bx, Continuous variables are represented by mean ± SD and categorical variables are shown as frequency (%).

## 4 DISCUSSION

In this study, B6.CCR5^−/−^ mice experienced less severe renal IRI than did wild‐type mice; comparatively, they had lower Cr concentrations, less tubular epithelial cell apoptosis, and less T‐cell and macrophage infiltration. When vascular endothelial cells are injured by ischaemia/reperfusion, vascular permeability and adhesion molecule expression increase, and the accumulation of leucocytes in the kidney accelerates. The role of T lymphocytes in this process is well known^20^, ^21^, ^22^, ^23^: when renal tubular cells are injured or stimulated in various ways, including activation by the complement system, expression of Toll‐like receptors increases, promoting the production of chemokines such as MCP‐1, CXCL8 and RANTES.^24^, ^25^ We confirmed the results of previous studies regarding T cells and their relationship with IRI in a B6.CCR5^−/−^ mouse model.^26^ Our results are consistent with a crucial role for T cells in the innate immune responses of both wild‐type and B6.CCR5^−/−^ mice. We presented for Figure S1 that is proposed mechanism of the association between CCR5, Th1‐type T cells and monocyte/macrophage.^20^, ^25^, ^26^ Here, we focused on kidney damage and its up‐regulation by CCR5, and explored the relevance and importance of macrophage infiltration.

Chemokines are 8‐10 kD cytokines that stimulate leucocyte migration and chemotaxis to sites of inflammation. The chemokine receptor CCR5 is mediated by RANTES, monocyte inflammatory protein 1α (CCL3) and MIP‐1β (CCL4), which are chemokine ligands produced in response to inflammation (Figure S2).^7^ Interestingly, in kidney transplant recipients with a CCR5‐Δ32 base pair deletion, kidney graft survival is longer than that in recipients with wild‐type CCR5, indicating the need for further studies to investigate the relationship between transplantation and CCR5 pathophysiology.^27^, ^28^ CCR5 inhibition protects against kidney damage in several animal models of kidney disease, and CCR5 antagonists reduce kidney damage by lessening the deposition of mononuclear cells in an experimental glomerular nephritis model.^29^ CCR5 may attenuate renal damage by reducing T‐cell deposition, as demonstrated in an acute kidney injury model.^26^ Based on animal models, when CXCR3 and its chemokine co‐receptor CCR5 are both inhibited, acute and chronic rejection of heart transplants is reduced through decreased T‐cell activity.^30^ Co‐expression of CXCR3 and CCR5 is prominent in macrophages, as shown in previous HIV studies.^7^, ^31^ Taken together, these results suggest that macrophages also play a crucial role in the action of chemokines that is likely similar to that of T cells.^32^ Therefore, in this study, we first observed crosstalk between CCR5 and other chemokines such as CXCR3 or CXCR4 (Figure 4). After inducing IRI, CCR5 co‐localized with CD11b^‐^positive cells, and consequently, the CXCR3 chemokine, which binds to macrophages or T lymphocytes,^33^ was expressed at a significantly lower level in B6.CCR5^−/−^ mice; however, CD11^+^/CXCR4^+^ expression was not significantly modulated. The difference in expression was more pronounced for CXCR3, suggesting that co‐signalling with CXCR3 may be important for CCR5 function.^30^

Next, to investigate the functional role of macrophage phenotype and CCR5 in IRI (Figure S3.), we compared heterogenic macrophage infiltration in wild‐type and B6.CCR5^−/−^ mice (Figure 5). As expected, macrophages in B6.CCR5^−/−^ mice, which had less injury, were highly expressed for the CD206^+^ M2 phenotype after IRI induction. In this study, we also showed that macrophage depletion with LC may reduce ischaemic acute kidney injury (AKI) in the context of CCR5 deficiency. Adoptive transfer of LPS‐treated RAW 264.7 macrophages, which constitutively express iNOS, reversed the functional protection against AKI in LC‐treated wild‐type and B6.CCR5^−/−^ mice (Figure 7). With LC‐induced macrophage depletion, adoptive transfer of M1 macrophages to B6.CCR5^−/−^ mice resulted in a significant increase in injury compared with that in mice treated with RAW 264.7 macrophages, or with RAW 264.7 macrophages and LPS (M1‐polarized). This suggests that M2 macrophages are expressed at higher levels in B6.CCR5^−/−^ mice and that their expression is down‐regulated by LC depletion, indicating that transferred M1 macrophages may promote an inflammatory response. Macrophages contribute to IRI and accelerate inflammation through various cytokines, mononuclear deposition and epithelial cell apoptosis. Macrophage depletion and repletion have been studied previously in IRI mouse and rat models,^2^, ^34^, ^35^, ^36^ and macrophages also contribute to long‐term fibrosis after IRI.^36^ In hypoxic kidney conditions, macrophages secrete pro‐angiogenic growth factors and pro‐inflammatory cytokines.^37^, ^38^ Macrophages respond to hypoxia by increasing chemokine secretion, which accelerates the recruitment and chemotaxis of macrophages and exacerbates hypoxia of the microenvironment itself.^37^, ^38^, ^39^ This is thought to be the main mechanism that attenuates damage in B6.CCR5^−/−^ mice.

When IRI occurs, immune cells are deposited and cytokine overproduction occurs because hypoxia is caused by a response to transcription factors that stimulate cytokines, including nuclear factor κB (NF‐κB), heat shock factor protein 1 and HIF‐1α.^38^, ^40^ Chemokines are also a major stimulant of chemotaxis, particularly when guiding neutrophils and M1 macrophages to the site of inflammation.^41^, ^42^ Chemokine receptor 2 (CCR2) promotes macrophage migration directly in IRI,^43^ but the effect of CCR5 on macrophage migration in IRI is not well understood. In this study, in vitro experiments were conducted to determine the relationship between the chemotactic effects of CCR5 and macrophages. Macrophages enhanced the secretion of pro‐inflammatory cytokines in tubular epithelial cells (TECs). Moreover, blockade of CCR5 and TAK779 inhibited the migration of macrophages (Figure 8). In recombinant CCR5‐treated proximal TECs, macrophage migration (Figure 8B) and iNOS expression increased (Figure 8C). These results show that co‐signalling of CCR5 and CXCR3 plays a major role in macrophage recruitment and migration through M1 macrophage activation, as well as in the Th1 response. We found that injury is alleviated by TAK779, which blocks both CCR5 and CXCR3. Co‐signalling of CXCR3 is important for the crosstalk between macrophages and CCR5, as shown in Figure 4. To further support these findings, we have provided the results for the supplement experiment for CCR5 regulation under T cell‐depleted conditions (Figures [Link], [Link], [Link], [Link], [Link], [Link]). Polarization of macrophages results in a switch in chemokine receptor expression, allowing macrophages to enter lymphatic vessels and to be actively drained to lymphoid tissues, which are the native T‐cell areas. Naive T cells interact with differently polarized macrophages and are activated, and T helper 1 (Th1) or 2 (Th2) effectors migrate to the peripheral tissues. IL‐12, which is a pro‐inflammatory molecule produced mainly by antigen‐presenting cells (APCs), plays a key role in this process. IL‐12 mainly activates natural killer cells and induces the differentiation of naïve CD4+ T lymphocytes to interferon‐gamma (IFN‐γ)‐producing Th1 effectors in cell‐mediated immune responses to inflammation, and the autocrine and paracrine effects of IFN‐γ activate APC recruitment and T‐cell activation. These supplementary experiments were conducted to explore which of the T cells and macrophages plays the major role in the association between CCR5 and kidney IRI. Based on the results presented in Figures [Link], [Link], [Link], [Link], [Link], [Link] and the main results, we have been suggested that both T cells and macrophages are important and influence each other and that CCR5 plays a pivotal role in IRI (Figure S3. Proposed mechanism of the association between CCR5 and macrophage polarization and M1/M2 transition). However, further experiments for the underlying mechanisms are needed, especially for the heterogeneity in macrophage expression via CD206 and arginase‐1 under T cell‐depleted conditions in CCR5 KO mice.

Previous studies have indicated a correlation between CCR5 expression on the surface of T cells and macrophages and cellular rejection in patients with biopsy‐proven acute rejection.^44^, ^45^ In this study, to evaluate tissue expression of p‐CCR5 in IRI, we performed IHC staining and morphometric analysis in pathologically confirmed ATN, but only in tissues from 25 patients, including kidney transplant recipients with no evidence of rejection and patients with glomerulonephritis. There was no difference in the renal survival rate in these patients based on a Kaplan‐Meier curve for the occurrence of end‐stage renal disease in accordance with morphometry positivity area (%)‐based grouping (lower than the median value of p‐CCR5 vs higher than the median value p‐CCR5; data not shown). However, tissue p‐CCR5‐positive areas increased with the severity of tubulitis and intimal arteritis (Table 1). We confirmed that inflammatory cells, including those with the CCR5 receptor for chemokine ligands in tubulitis and endothelialitis, correspond to the distribution of appropriate ligands.

In summary, we identified the role of CCR5, which modulates inflammation and immunity, in macrophage induction of IRI. We showed that CCR5 blockade attenuated IRI via the macrophage heterogenic signalling pathway, irrespective of the anti‐inflammatory effects of T cells. Modulation of the CCR5 pathway could thus prove useful as a new therapy for ischaemic AKI.

## CONFLICT OF INTEREST

The authors confirm that there are no conflicts of interest.

## AUTHOR CONTRIBUTIONS

KD Yoo, SH Yang and YS Kim conducted most experiments, analysed the results and wrote most of the manuscript. SH Yang and KD Yoo conducted the experiments on the role of CCR5 and macrophages in kidney IRI. S Lee, JE Kim, KH Kim and SH Yang conducted the experiments on CCR5 and macrophage heterogeneity. RH Cha, JS Lee, and DK Kim conceived the idea for the project and wrote the manuscript with KD Yoo and SH Yang.

## Supporting information

Appendix S1

Figure S1

Figure S2

Figure S3

Figure S4

Figure S5

Figure S6

Figure S7

Figure S8

Figure S9

Figure S10

## Data Availability

The data that support the findings of this study are available from the corresponding author, SH Yang, upon reasonable request.
