# Supplementary material for: Chemokine receptor 5 blockade modulates macrophage trafficking in renal ischaemic‐reperfusion injury
Source: J Cell Mol Med. 2020 Mar 30;24(10):5515–27. doi: 10.1111/jcmm.15207 (PMC7214177; doi:10.1111/jcmm.15207)
Supplement: Supplementary file 1 — Appendix S1 [file JCMM-24-5515-s001.docx]

***Supplementary materials table and figures of contents***

Induction of renal IRI

Histology analysis

Confocal microscopy

Quantitative real-time PCR

Western blot analysis and cytokine assays

Flow cytometry analysis

In vitro assay in ischemia-induced hypoxic conditions

RAW 264.7 macrophage culture and adoptive transfer into macrophage-depleted mice

Bone marrow-derived macrophage isolation, culture, and in vitro chemotactic migration assay

Human clinical sample study

Statistical analysis

Supplementary figure legends

***Induction of renal ischemic reperfusion injury***

The experimental study protocols used in our study have been previously described [[1-4](#_ENREF_1)]. Mice were anesthetized by intraperitoneal injection of pentobarbital sodium (Nembutal, 50 mg/kg body weight; Abbott, Wiesbaden, Germany) and ketamine (100 mg/kg body weight). After bilateral flank incision, both renal pedicles were dissected and clamped with a microvascular clamp (Roboz Surgical Instrument, Gaithersburg, MD, USA) for 30 min [[1](#_ENREF_1),[2](#_ENREF_2),[5](#_ENREF_5)]. During the procedure, 2 mL of sterile saline at 40 °C (1 mL during ischemia and 1 mL during reperfusion) were introduced into the peritoneal cavity. Body temperatures were recorded and maintained at 37 °C during renal IRI. Following clamp removal, wounds were sutured, and appropriate reperfusion was confirmed by visual inspection. Sham-operated mice underwent the same surgical procedures, except for the timing of renal pedicle timing. Mice were killed 2 days (48 h) after reperfusion, and blood samples were obtained from tail veins. Renal function was evaluated by measuring serum creatinine (Cr) concentrations using the modified Jaffé rate reaction and auto-analyzer (Hitachi Chemical, Osaka, Japan). Mice were inducing T cell depletion by intraperitoneal injection of CD3 antibody (abcam, 500μ) at 2day before, and just before IRI , conducted in supplement experiments (Figure S4 to S9). Five to eight mice were used in each group, and three independent experiments were performed for each procedure (**Fig S4.**).

***Histology analysis***

Mice were sacrificed 2 days (48 h) after IRI and kidneys were harvested after exsanguination as previously described [[1](#_ENREF_1),[2](#_ENREF_2)]. Tissue samples were fixed with 10 % buffered formalin and paraffin embedding. Tissue sections (4 µm thick) were prepared from paraffin blocks and stained with periodic acid-Schiff reagent. The degree of tubular injury was assessed by a renal histologist who was blinded to group assignment. Cell loss and necrosis were graded on five levels (1: <10 %; 2: 10–25 %; 3: 25–50 %; 4: 50–75 %; 5: >75 %), based on the percentage of injured tubules in the cortex and two different lesions, respectively.

Stained slides were examined using an Olympus inverted microscope (Olympus Imaging America, Center Valley, CA)[[2](#_ENREF_2)]. Injured tubules were counted based on the proportion of necrotic tubules and tubular casts relative to total number of tubules as described in previously [[6](#_ENREF_6)]. Cell loss and necrosis were graded in five levels and presented as histomorphological scores based on PAS staining.

***Confocal microscopy***

Immunofluorescence was performed using an LSM 510 Meta laser confocal microscope (Carl Zeiss, Jena, Germany). Paraffin-embedded samples were collected and cut into 4-µm slices. Slices were then deparaffinized and hydrated using xylene and ethanol, which were then stained with primary antibodies in a blocking reagent overnight at 4°C. The following antibodies were used: mouse CCR5 (Abcam, Cambridge, UK), CD3 (BD Biosciences, Franklin Lakes, NJ, USA), rat F4/80 (Abcam, Cambridge, UK), rabbit myeloperoxidase (MPO) (Abcam, Cambridge, UK), CD11b (Abcam, Cambridge, UK), CXCR3 (Abcam, Cambridge, UK), CXCR4 (Abcam, Cambridge, UK), and CD206 (Santa Cruz Biotechnology, Dallas, USA). Alexa FluorH 488-conjugated goat anti-rabbit antibody (Molecular Probes, Eugene, OR, USA), Alexa FluorH 555-conjugated anti-rat antibody, and Alexa FluorH 555-conjugated anti-mouse antibody were used as secondary antibodies. All sections were washed and incubated for an additional 5 min with 49,6-diamidino-2-phenylindole (DAPI) for counterstaining. Primary antibodies were omitted from sections in the negative control. In another set of experiments, the kidneys were snap-frozen in Optimal Cutting Temperature (OCT) embedding medium (Miles, Elkhart, IN, USA), cooled to −80 °C, and cut into 5-μm-thick sections using a cryostat (Leica, Heidelberger, Germany). Frozen sections were fixed for 10 min in cold acetone.

***Quantitative real-time PCR***

Total RNA was extracted from the harvested renal tissues 48 h after induction of IRI. Cytokine mRNA concentrations were assayed using real-time PCR.

In brief, total RNA was isolated from kidneys using an RNeasyH kit (Qiagen GmBH, Hilden, Germany), and 1 μg of the total RNA was reverse-transcribed to cDNA using oligo-d(T) primers and AMV-RT Taq polymerase ( category number. A3500; Promega, Madison, WI, USA).

Real-time PCR was performed using assay-on-demand TaqManH probes and primers for TNF-α, interferon-γ, MCP-1, interleukin (IL)-10, CCL3, CCL4, CCL5, CCL8, iNOS, arginase-1, and glyceraldehyde 3-phosphate dehydrogenase (GAPDH) (Applied Biosystems; Thermo Fisher Scientific, Inc.), and an ABI PRISM 7500 Sequence Detection System (Applied Biosystems). The mRNA levels for each cytokine were normalized using GAPDH mRNA expression. Primer sequences were as follows: moust TNF-α, forward 5′-CGGCACAGTCATTGAAAGCCTA-3′ and reverse 5′-GTTGCTGATGGCCTGATTGTC-3′; interferon-γ ; mouse MCP-1, forward 5′-GCTCAGCCAGATGCAGTTAA-3′ and reverse 5′-TCAAAAACAGTGGTTCGAGTTCT-3′; mouse IL-10, forward 5′-ATAACTGCACCCACTTCCCA-3′ and reverse 5′-TGGACCATCTTCACTACGGG-3′; iNOS, forward 5’-CGAAACGCTTCACTTCCAA-3’ and reverse 5’-AACACGGCAGTGGCTTTAACC-3’; arginase-1, forward 5’- AACACGGCAGTGGCTTTAACC-3’ and reverse 5’-GGTTTTCATGTGGCGCATTC-3’ ; mouse CCR5, forward 5′-TTGTCTACTTTCTCTTCTGG-3′ and reverse 5′-ATCGGGTATAGACTGAGC-3′; mouse GAPDH, forward 5′-CACCAAGAGCAGCCACCTCA-3′ and reverse 5′-CGGGACACTGGTACGGCTTC-3′.

***Western blotting analysis and cytokine assays***

The effects of macrophages on protein levels in B6.CCR5^-/-^ mice were analyzed using western immunoblotting and cytokine assays. Arginase-1 and β-actin (Sigma-Aldrich, St. Louis, MO, USA), primary antibodies against CD206, P-CCR5, CCR5, iNOS (Abcam, Cambridge, UK), and HIF-1α (Novus Biologicals, Colorado, USA) were used in the western blot analysis. Equal amounts (80 μg) of extracted proteins were separated by 10 % sodium dodecyl sulfate-polyacrylamide gel electrophoresis and transferred onto Immobilon-FL 0.4-μm polyvinylidene difluoride membranes (Millipore, Bedford, MA). Anti-rabbit IgG (Vector Laboratories, Burlingame, CA) was used as the secondary antibody, and blots were developed using Super Signal West Pico Chemi-luminescent Substrate (Pierce, Woburn, MA, USA). Densitometric analyses were performed using ImageJ software (National Institutes of Health, Bethesda, MD, USA, https://imagej.nih.gov/ij/).

ELISA and a multiplex cytokine bead array system (Bio-Plex; Bio-Rad) were used to assay cytokines such as IL-4, IL-6, IL-8, IL-10, IL-13, and interferon-γ following the manufacturer’s instructions.

***Flow cytometry analysis***

For quantitative flow cytometry analysis, intrarenal mononuclear cells were isolated from mouse kidneys, with homogenates obtained using a StomacherH 80 Biomaster (Seward, Worthing, Sussex, UK). Single-cell suspensions were obtained by passing the tissue through a 40-μm cell strainer. Kidneys were resuspended in 36 % Percoll (Amersham Pharmacia Biotech, Piscataway, NJ, USA) and overlaid onto 72 % Percoll. After centrifuging for 30 min at 1000 × *g* and 25 °C, renal mononuclear cells were isolated from the interface and incubated with mouse monoclonal antibodies directly conjugated to CD3, CD44, and CCR5 (BD Biosciences, Bedford, MA, USA). For intracellular cytokine staining, 5 × 10^5^ mononuclear cells were plated in RPMI 1640 medium supplemented with 10 % fetal calf serum, 100 U/mL penicillin, and 100 μg/mL streptomycin (Gibco, Carlsbad, CA, USA), and then activated with phorbol 12-myristate 13-acetate (PMA, 100 ng/mL, Sigma-Aldrich) and ionomycin (500 ng/mL, Sigma-Aldrich) in the presence of Brefeldin A (10 μg/mL, BD Pharmingen, San Diego, CA, USA) at 37 °C for 4 h. After activation, T cells were permeabilized with BD Cytofix/Cytoperm solution according to the manufacturer’s instructions (BD Pharmingen San Diego, CA, USA), stained with antibodies, and fixed with 1 % paraformaldehyde. Fluorescence signals were detected using a FACSCalibur instrument, and cell frequencies were analyzed using CellQuest (BD Biosciences, Bedford, MA, USA) and FlowJo software (version 10.0.7; FlowJo LLC, Ashland, OR, USA).

***In vitro assay in ischemia-induced hypoxic conditions***

To demonstrate the role of CCR5 inhibition in a hypoxic state, we conducted *in vitro* experiments using tubular epithelial cell lines. Initially, we evaluated the expression of CCR5, HIF-1α, and p-CCR5 in HK-2 cells in normoxic conditions. Simultaneously, we cultured HK-2 cells in hypoxic conditions (21 % O_2_, 5 % CO_2_, and 74 % N_2_) with TAK779 (0 μM, 20 μM, and 40 μM) (Sigma-Aldrich, Saint Louis, MO, USA). TAK779 is a CCR5 antagonist with a beneficial effect against human immunodeficiency virus [[7](#_ENREF_7)]. After 6 h under these conditions, cells were harvested to evaluate the expression of p-CCR5 and HIF-1α as an acute response to hypoxia.

***RAW 264.7 macrophage culture and adoptive transfer into macrophage-depleted mice***

RAW 264.7 macrophages cells are very well known as an appropriate experimental model of macrophage immune response. The biological properties of these RAW 264.7 macrophages have been recently discovered [[8](#_ENREF_8)]. RAW 264.7 cells are monocyte / macrophage-like cells derived from Abelson leukemia virus transgenic cell line derived from BALB/C mice. Upon LPS stimulation, RAW 264.7 cells show nitric oxide (NO) production and phagocytosis with a phenotype of M1 macrophage [[9](#_ENREF_9)]. RAW 264.7 macrophages were cultured with or without LPS (100 ng/mL) for 16 hr cultivation (Figure 7A) as described previously [[10](#_ENREF_10),[11](#_ENREF_11)]. The concentrations of IFN-γ and IL-10 in supernatant samples were determined using the Bio-Plex Pro system (Bio-Rad Laboratories), an immunoassay system formatted on magnetic beads, for confirming M1 macrophage tendency. The cultured macrophages (1 × 10^8^ cells) were administered intraperitoneally 1 h prior to ischemia/reperfusion surgery [[10](#_ENREF_10),[11](#_ENREF_11)]. Macrophages were depleted 6 days before induction of IRI using LC.

Blood samples were collected from tail veins 48 h after induction of ischemic damage. Mice were sacrificed 48 h after reperfusion. Five to six mice were used for each group, and three independent experiments were performed for each procedure.

***Bone marrow-derived macrophage isolation, culture, and in vitro chemotactic migration assay***

Femurs of wild-type and B6.CCR5^-/-^ mice were obtained, bone marrow cells were isolated, and the cells were dissociated in bone marrow-derived macrophages (BMDM) growth medium. Fresh BMDM growth medium was supplied again on the third day of culture [[12](#_ENREF_12),[13](#_ENREF_13)], and BMDM formation was evaluated on the seventh day[[12](#_ENREF_12)]. Finally, macrophages were stimulated to analyze M1 macrophage polarization using LPS [[13](#_ENREF_13)] (Sigma-Aldrich, Saint Louis, MO, USA) and M2 macrophage polarization using recombinant mouse IL-4 protein[[13](#_ENREF_13)] (R&D Systems, MN, USA). A Boyden chamber assay with wells separated by a polycarbonate filter and pores small enough to allow only active passage from cells (8-μm porous membrane, Cell Biolabs, San Diego, USA) was used in the *in vitro* chemotactic migration assay [[14](#_ENREF_14),[15](#_ENREF_15)]. Twelve h prior to initiation of the migration assay, fresh medium composed of 15 % FBS and DMEM/F12 was added. A chamber assay with a fibronectin-coated membrane standing on fibronectin (0.1 % solution, 1 mg/mL) was used (Sigma, F1141-1MG) for about 4–5 h after the membrane dried at room temperature. After TECs were cut and counted, stabilized cells were placed in 1.0 × 10^6^/mL 15 % complete media for 1 h at 37 °C. Primary cultured proximal TECs of wild-type mice were seeded at the bottom. TECs were isolated from kidney tissues of wild-type and B6.CCR5-/- mice. Upon dissection of cortices, fresh specimens were minced and dissolved with Hank's balanced salt solution (HBSS) containing 3 mg/mL collagenase (Sigma-Aldrich, St. Louis, MO, USA). Following centrifugation for 5 min at 500 × *g*, TECs were isolated.

After 30 min, recombinant CCR5 (1 µg/mL) and CCR5 inhibitor (TAK779) were added (20 μM), and the cells were incubated for an additional 30 min. BMDMs were injected with LPS and stored for 4–5 h at 37 °C to preserve humidity before being placed into an incubator. Next, BMDM from wild-type and B6.CCR5^-/-^ mice were co-cultured in the upper chamber. After 3 h, cells were inverted and stained with DAPI. After incubation, the chamber was removed, and the membrane was stained. The membrane was then washed in distilled water, and the chamber membrane was turned, attached to a glass cell, and cells were counted [[14](#_ENREF_14),[15](#_ENREF_15)].

***Human clinical sample study***

The Institutional Review Board of Seoul National University Hospital approved the protocols associated with renal biopsy samples from patients with transplants (n = 14) or glomerulonephritis (n = 11) and from normal controls (n = 6). Normal controls showed minimal nonspecific changes in glomeruli or tubules. Normal control tissues were obtained using kidney tissues from patient renal biopsy samples without structural changes. We collected unstained slides from 14 patients who were clinically diagnosed with delayed graft function, defined by dialysis maintained for 2 weeks after transplantation. Specimens with biopsy-proven ATN without evidence of cellular rejection were used to determine whether p-CCR5 had a detrimental effect on IRI in patients who had undergone transplantation. Moreover, 11 patients with glomerulonephritis were also examined after ATN confirmation. The number of p-CCR5 cells was measured using IHC staining and morphometric analysis. The number of p-CCR5 cells was counted in five randomly selected fields based in biopsied tissues. For each kidney sample, five fields were viewed at 100× magnification under a light microscope, and the p-CCR5 positivity area (%) was evaluated using a morphometric system (Qwin 3, Leica, Netherlands).

***Statistical analysis***

Most analyses and calculations were performed using IBM SPSS Statistics V21.0 (IBM Corporation, Armonk, NY, USA) and Prism version 5 (GraphPad Software, La Jolla, CA, USA). The results were analyzed using Kruskal-Wallis nonparametric testing for multiple comparisons, Mann-Whitney U testing, one-way ANOVA followed by Tukey’s post-hoc analysis, and two-way ANOVA followed by Bonferroni post-hoc analysis. P values less than 0.05 were considered statistically significant.

***Supplementary figure legends***

Figure S1. Proposed mechanism of the association between CCR5, Th1-type T cells, and monocyte/macrophage

Figure S2. The mRNA levels of chemokine receptor ligands after inducing IRI.

Figure S3. Proposed mechanism of the association between CCR5 and macrophage polarization and M1/M2 transition

Figure S4. Detailed method for induction of renal ischemic reperfusion injury with T cell depletion.

Figure S5. The role of CCR5 regulation on T cell depleted condition in kidney IRI by laboratory data.

Figure S6. The role of CCR5 regulation on T cell depleted condition in kidney IRI by histologic data.

Figure S7. The crosstalk of macrophage and CCR5 after IRI in T cell depleted WT mice using confocal microscopy.

Figure S8. The crosstalk of macrophage and CXCR3 after IRI in T cell depleted situation using confocal microscopy.

Figure S9. The crosstalk of macrophage and CXCR4 after IRI in T cell depleted situation using confocal microscopy.

Figure S10. Detailed method for Boyden chamber migration assay.

**REFERENCE**

1. **Yang SH LJ, Jang HR, Cha RH, Han SS, Jeon US, Kim DK, Song, J, Lee DS, Kim YS.** Sulfatide-reactive natural killer T cells abrogate ischemia-reperfusion injury. *J Am Soc Nephrol*. 2011; 22: 1305-14.

2. **Lee JP, Yang SH, Lee HY, Kim B, Cho JY, Paik JH, Oh YJ, Kim DK, Lim CS, Kim YS.** Soluble epoxide hydrolase activity determines the severity of ischemia-reperfusion injury in kidney. *PloS one*. 2012; 7: e37075.

3. **An JN, Yang SH, Kim YC, Hwang JH, Park JY, Kim DK, Kim JH, Kim DW, Hur DG, Oh YK, Lim CS, Kim YS, Lee JP.** Periostin induces kidney fibrosis after acute kidney injury via the p38 MAPK pathway. *American journal of physiology Renal physiology*. 2019; 316: F426-F37.

4. **Lee JW, Bae E, Kwon SH, Yu MY, Cha RH, Lee H, Kim DK, Lee JP, Ye SK, Yoo JY, Park DJ, Kim YS, Yang SH.** Transcriptional modulation of the T helper 17/interleukin 17 axis ameliorates renal ischemia-reperfusion injury. *Nephrology, dialysis, transplantation : official publication of the European Dialysis and Transplant Association - European Renal Association*. 2019; 34: 1481-98.

5. **MARK D. OKUSA JL, TIMOTHY MACDONALD, AND LIPING HUANG.** Selective A2A adenosine receptor activation reduces ischemia-reperfusion injury in rat kidney. *American journal of physiology Renal physiology*. 1999.

6. **HR J.** Early exposure to germs modifies kidney damage and inflammation after experimental ischemia-reperfusion injury. *American journal of physiology Renal physiology*. 2009.

7. **Alkhatib G.** The biology of CCR5 and CXCR4. *Current opinion in HIV and AIDS*. 2009; 4: 96-103.

8. **Taciak B, Bialasek M, Braniewska A, Sas Z, Sawicka P, Kiraga L, Rygiel T, Krol M.** Evaluation of phenotypic and functional stability of RAW 264.7 cell line through serial passages. *PloS one*. 2018; 13: e0198943.

9. **Tang PM, Nikolic-Paterson DJ, Lan HY.** Macrophages: versatile players in renal inflammation and fibrosis. *Nature reviews Nephrology*. 2019; 15: 144-58.

10. **Nishida M, Okumura Y, Fujimoto S, Shiraishi I, Itoi T, Hamaoka K.** Adoptive transfer of macrophages ameliorates renal fibrosis in mice. *Biochemical and biophysical research communications*. 2005; 332: 11-6.

11. **Ricardo SD, van Goor H, Eddy AA.** Macrophage diversity in renal injury and repair. *The Journal of clinical investigation*. 2008; 118: 3522-30.

12. **Trouplin V, Boucherit N, Gorvel L, Conti F, Mottola G, Ghigo E.** Bone marrow-derived macrophage production. *Journal of visualized experiments : JoVE*. 2013: e50966.

13. **Ying W, Cheruku PS, Bazer FW, Safe SH, Zhou B.** Investigation of macrophage polarization using bone marrow derived macrophages. *Journal of visualized experiments : JoVE*. 2013.

14. **Chen CY, Hsiau KC, Chung CA.** Measurement of chondrocyte chemotaxis using a Boyden chamber: a model of receptor-mediated cell migration combined with cell sedimentation. *Math Med Biol*. 2013; 30: 213-39.

15. **Thomsen R, Lade Nielsen A.** A Boyden chamber-based method for characterization of astrocyte protrusion localized RNA and protein. *Glia*. 2011; 59: 1782-92.
