# Supplementary figures and images for: Chemokine receptor 5 blockade modulates macrophage trafficking in renal ischaemic‐reperfusion injury
Source: J Cell Mol Med. 2020 Mar 30;24(10):5515–27. doi: 10.1111/jcmm.15207 (PMC7214177; doi:10.1111/jcmm.15207)

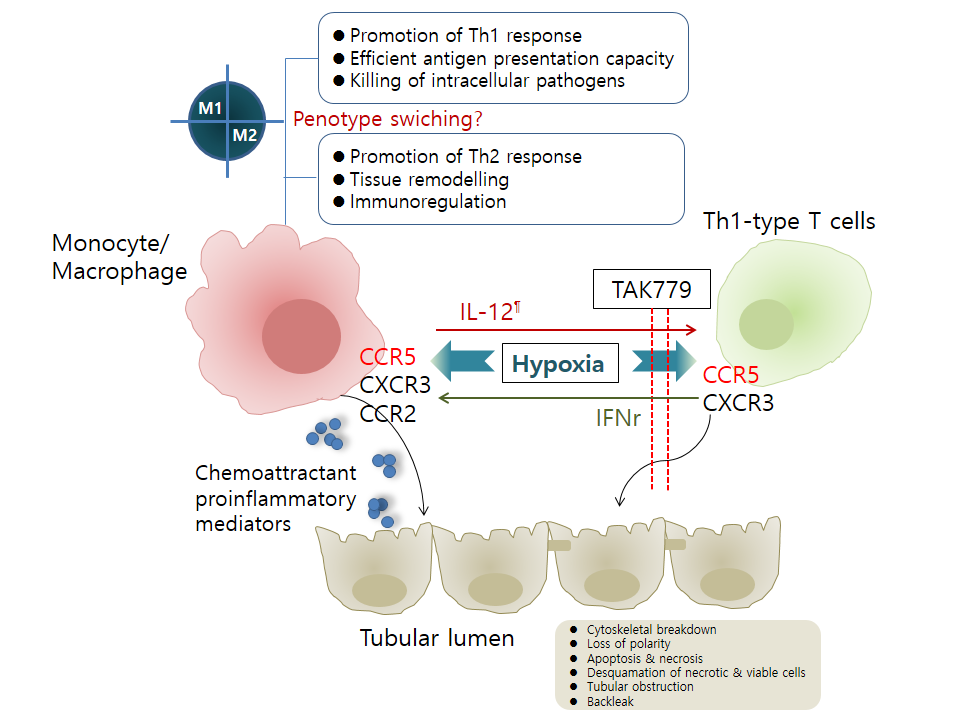

Supplement: Supplementary file 2 — Figure S1 [file JCMM-24-5515-s002.tif]

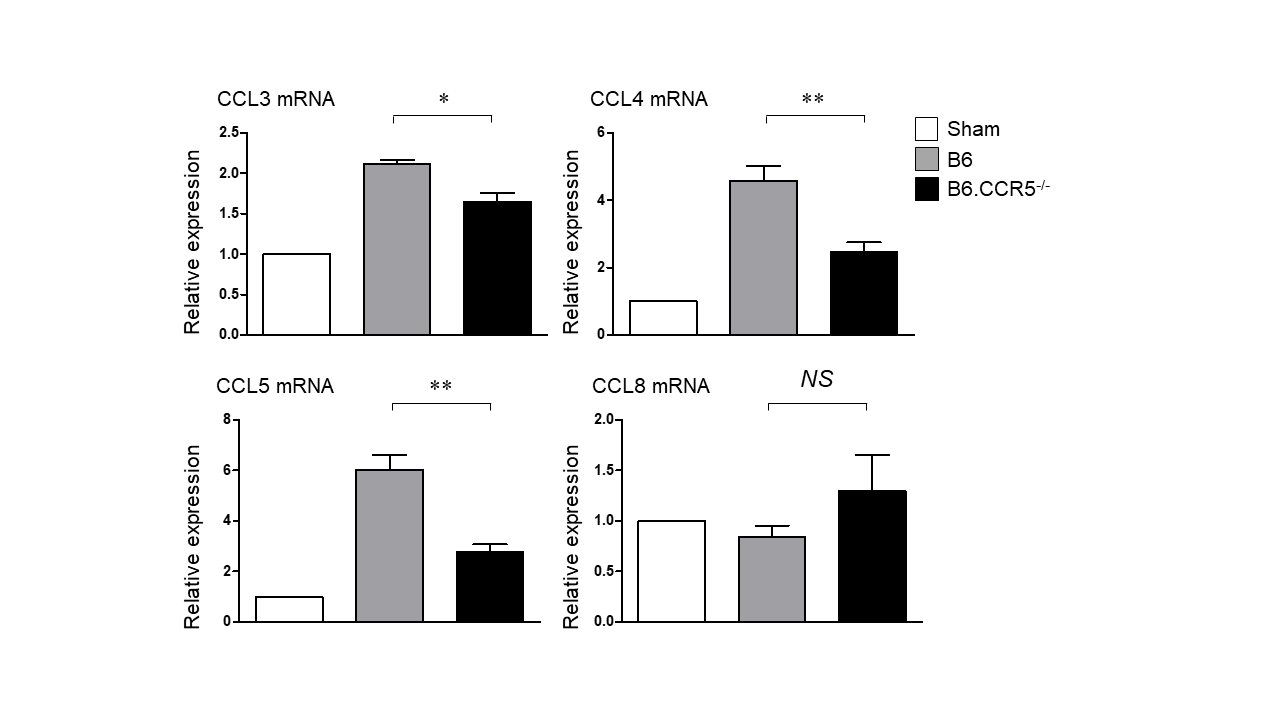

Supplement: Supplementary file 3 — Figure S2 [file JCMM-24-5515-s003.tif]

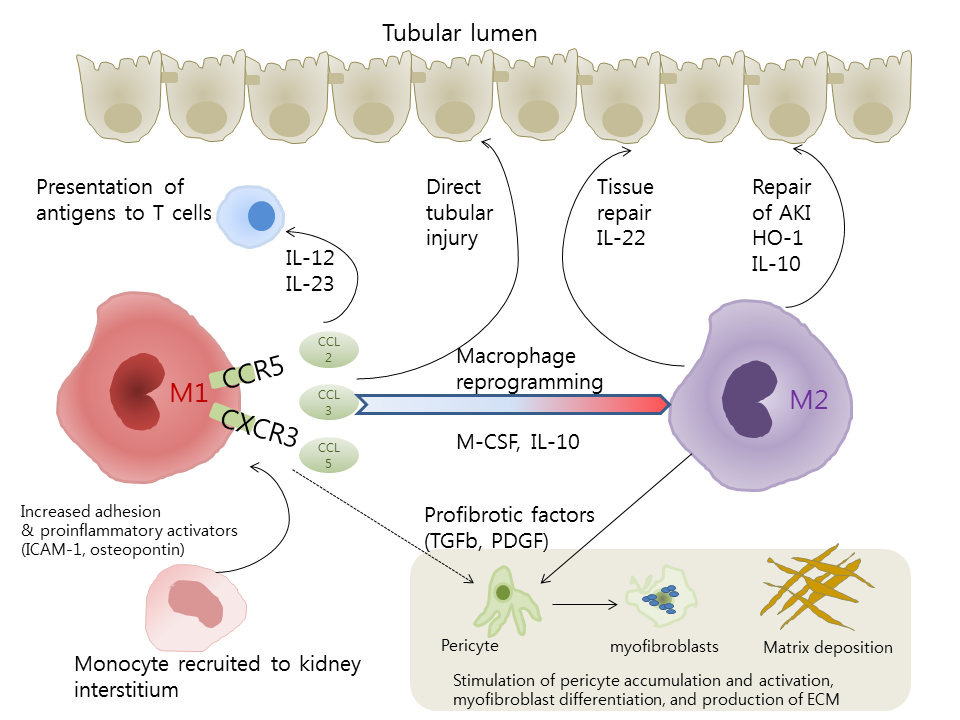

Supplement: Supplementary file 4 — Figure S3 [file JCMM-24-5515-s004.tif]

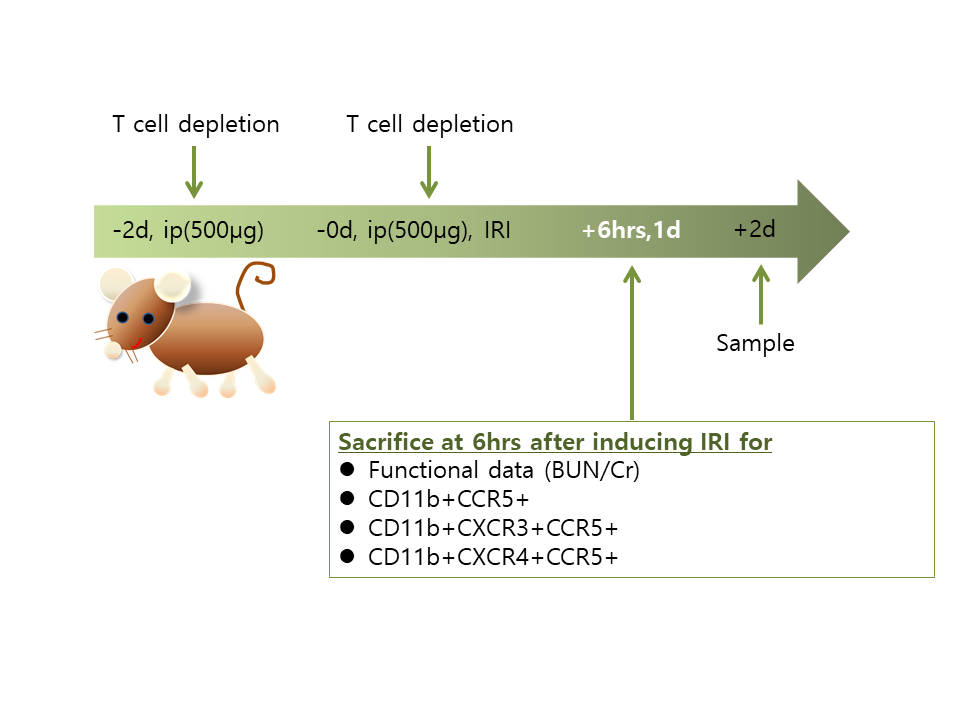

Supplement: Supplementary file 5 — Figure S4 [file JCMM-24-5515-s005.tif]

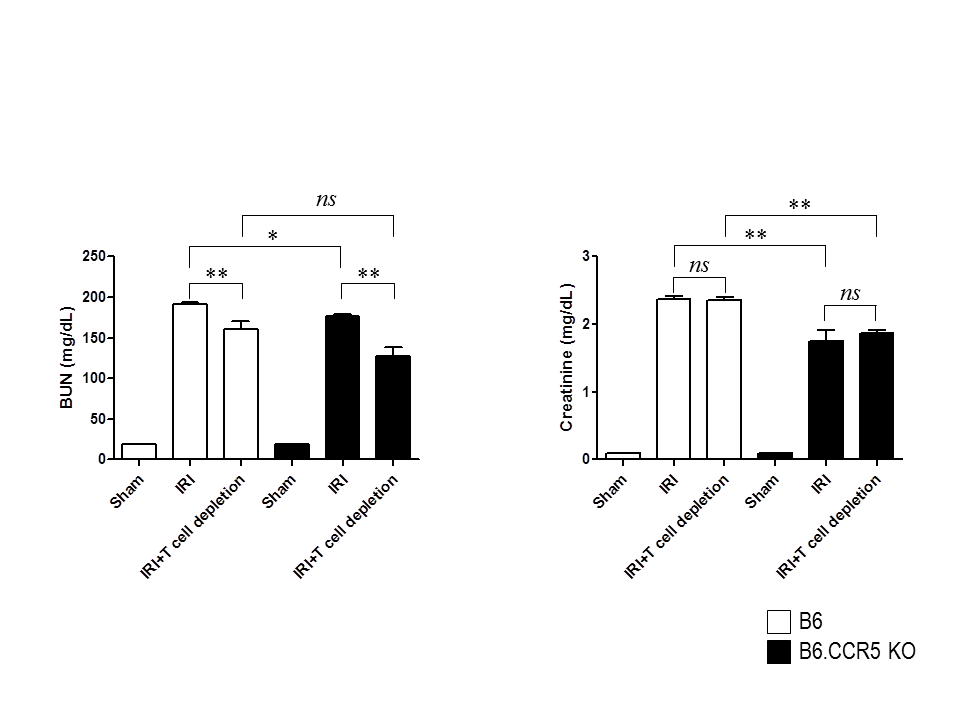

Supplement: Supplementary file 6 — Figure S5 [file JCMM-24-5515-s006.tif]

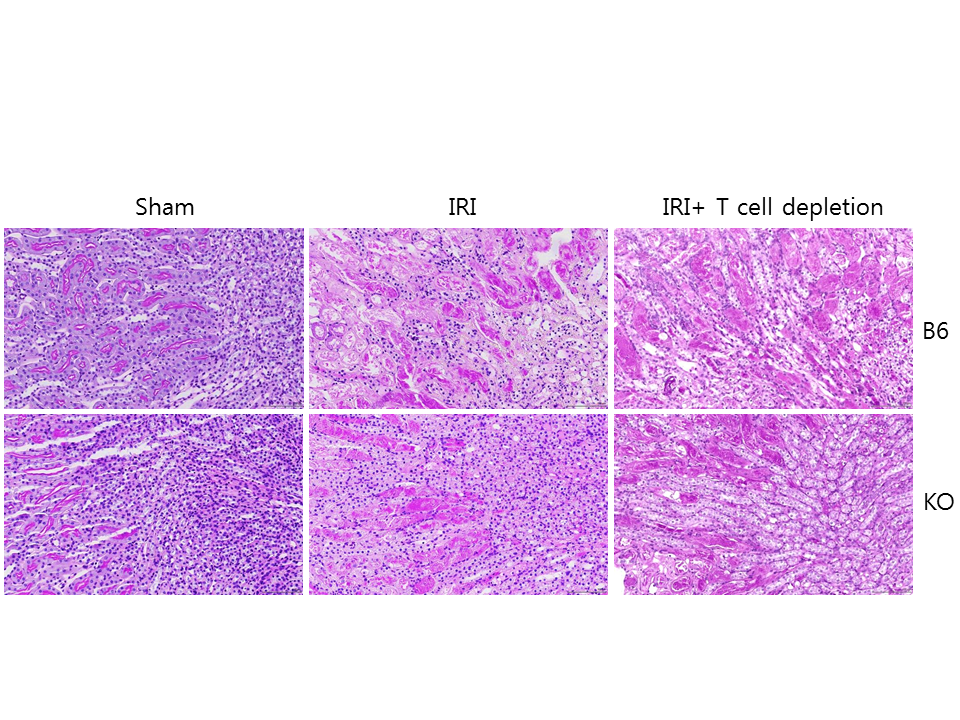

Supplement: Supplementary file 7 — Figure S6 [file JCMM-24-5515-s007.tif]

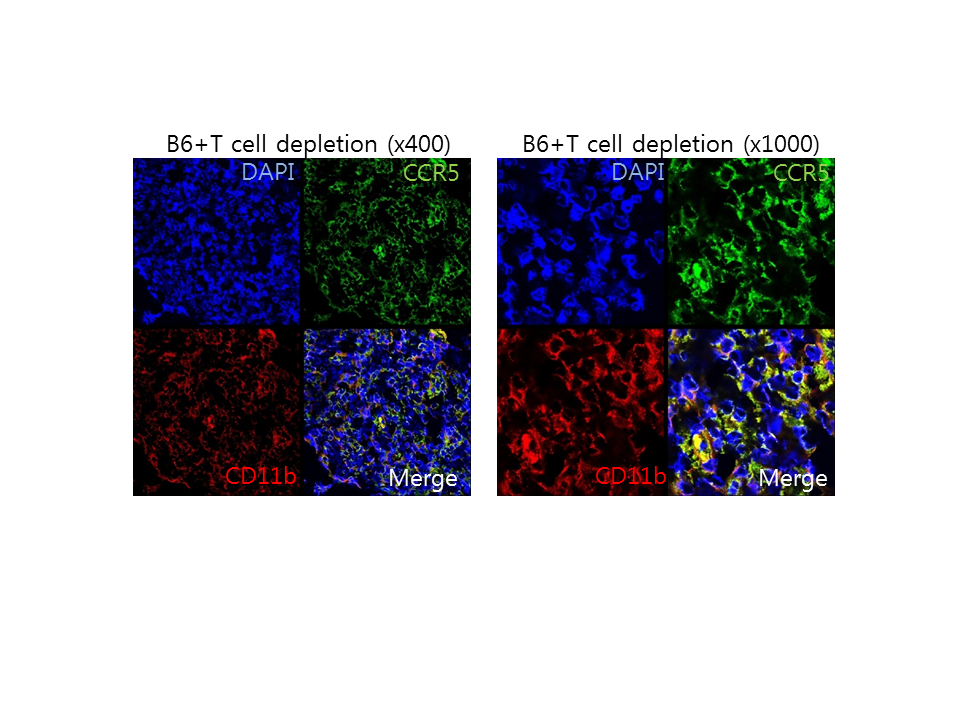

Supplement: Supplementary file 8 — Figure S7 [file JCMM-24-5515-s008.tif]

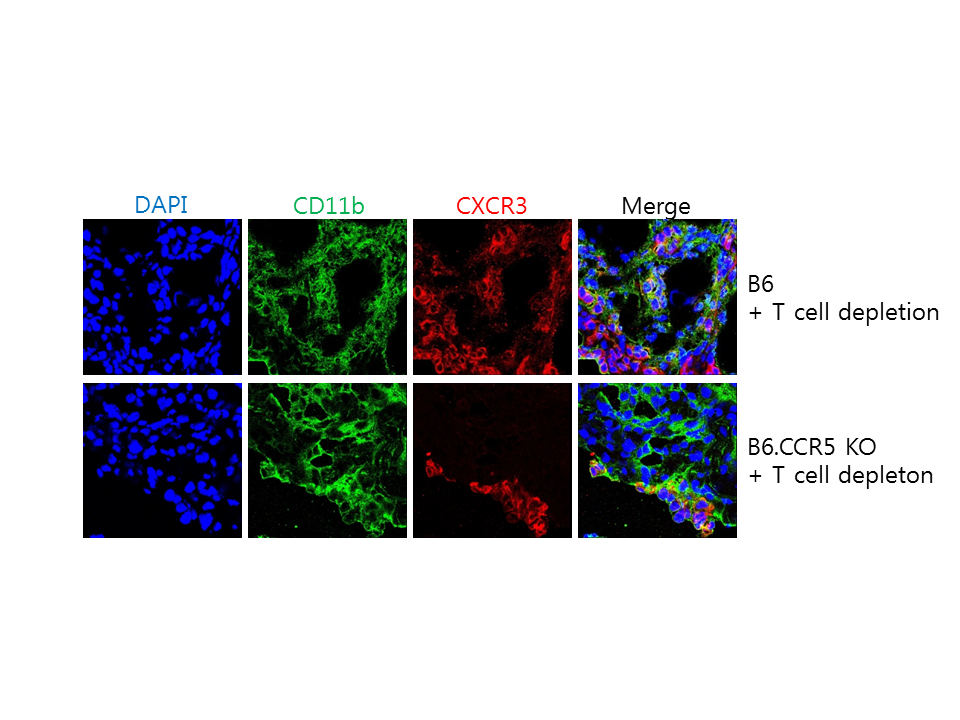

Supplement: Supplementary file 9 — Figure S8 [file JCMM-24-5515-s009.tif]

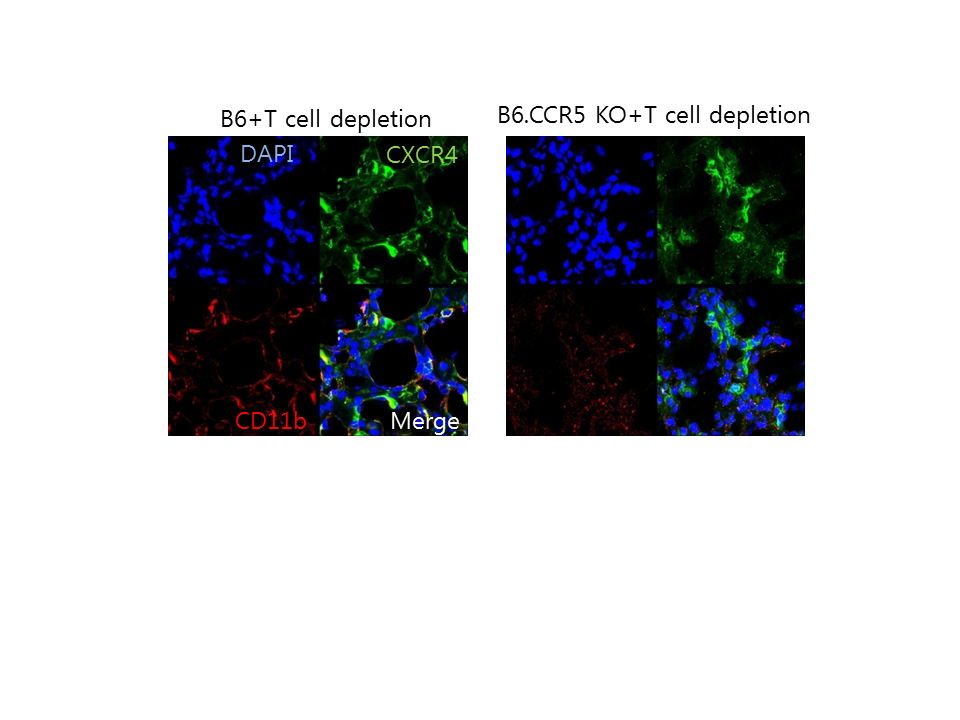

Supplement: Supplementary file 10 — Figure S9 [file JCMM-24-5515-s010.tif]

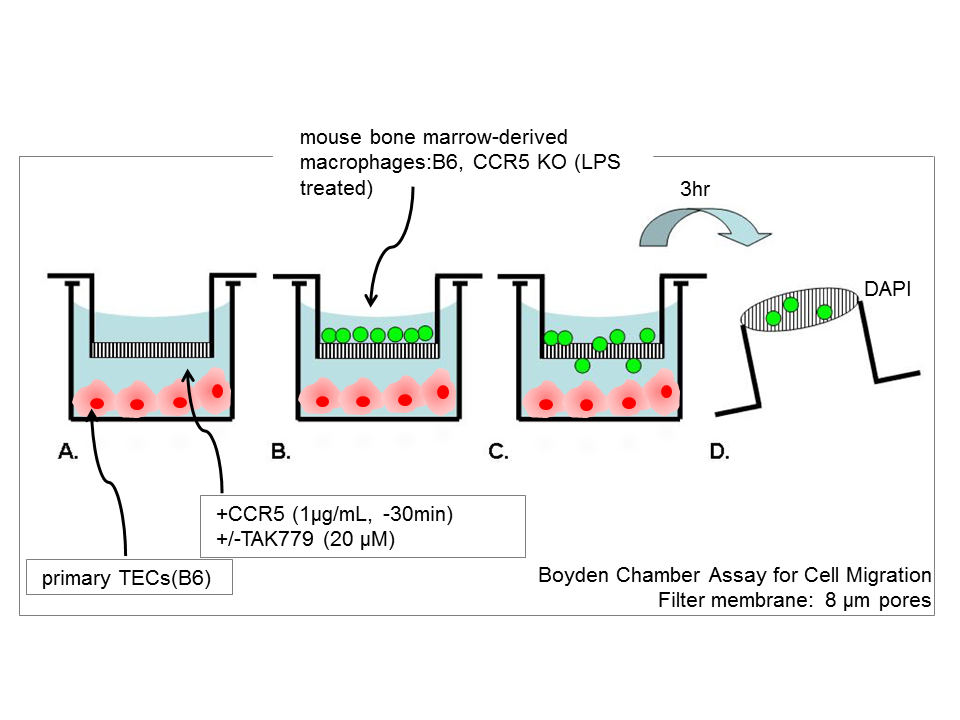

Supplement: Supplementary file 11 — Figure S10 [file JCMM-24-5515-s011.tif]
